# *Leishmania donovani* Nucleoside Hydrolase (NH36) Domains Induce T-Cell Cytokine Responses in Human Visceral Leishmaniasis

**DOI:** 10.3389/fimmu.2017.00227

**Published:** 2017-03-07

**Authors:** Micheli Luize Barbosa Santos, Dirlei Nico, Fabrícia Alvisi de Oliveira, Aline Silva Barreto, Iam Palatnik-de-Sousa, Eugenia Carrillo, Javier Moreno, Paula Mello de Luca, Alexandre Morrot, Daniela Santoro Rosa, Marcos Palatnik, Cristiane Bani-Corrêa, Roque Pacheco de Almeida, Clarisa Beatriz Palatnik-de-Sousa

**Affiliations:** ^1^Laboratório de Biologia Molecular, Hospital Universitário, Departamento de Medicina, Universidade Federal de Sergipe (HU-UFS), Aracaju, Sergipe, Brazil; ^2^Laboratório de Biologia e Bioquímica de Leishmania, Departamento de Microbiologia Geral, Instituto de Microbiologia Paulo de Góes, Universidade Federal do Rio de Janeiro (UFRJ), Rio de Janeiro, Rio de Janeiro, Brazil; ^3^Laboratório de Biometrologia, Programa de Pós-Graduação em Metrologia, Pontifícia Universidade Católica do Rio de Janeiro, Rio de Janeiro, Rio de Janeiro, Brazil; ^4^WHO Collaborating Centre for Leishmaniasis, Instituto de Salud Carlos III, Centro Nacional de Microbiologia, Madrid, Comunidad de Madrid, Spain; ^5^Laboratório de Imunoparasitologia, Instituto Oswaldo Cruz (IOC), Rio de Janeiro, Rio de Janeiro, Brazil; ^6^Laboratório de Imunologia Integrada, Departamento de Imunologia, Instituto de Microbiologia Paulo de Góes, Universidade Federal do Rio de Janeiro (UFRJ), Rio de Janeiro, Rio de Janeiro, Brazil; ^7^Faculdade de Medicina, Instituto de Investigação em Imunologia, Universidade de São Paulo (USP), São Paulo, Brazil; ^8^Laboratório de Vacinas experimentais, Departamento de Microbiologia, Imunologia e Parasitologia, Universidade Federal de São Paulo (UNIFESP), São Paulo, São Paulo, Brazil; ^9^Laboratório de Imunohematologia, Faculdade de Medicina, Hospital Universitário Clementino Fraga-Filho, Universidade Federal do Rio de Janeiro (UFRJ), Rio de Janeiro, Rio de Janeiro, Brazil; ^10^Departamento de Morfologia, Universidade Federal de Sergipe (HU-UFS), Aracaju, Sergipe, Brazil

**Keywords:** human visceral leishmaniasis, nucleoside hydrolase, recombinant domains, T cell epitopes, epitope vaccine design, *Leishmania donovani*, *Leishmania infantum chagasi*

## Abstract

Development of immunoprotection against visceral leishmaniasis (VL) focused on the identification of antigens capable of inducing a Th1 immune response. Alternatively, antigens targeting the CD8 and T-regulatory responses are also relevant in VL pathogenesis and worthy of being included in a preventive human vaccine. We assessed in active and cured patients and VL asymptomatic subjects the clinical signs and cytokine responses to the *Leishmania donovani* nucleoside hydrolase NH36 antigen and its N-(F1), central (F2) and C-terminal (F3) domains. As markers of VL resistance, the F2 induced the highest levels of IFN-γ, IL-1β, and TNF-α and, together with F1, the strongest secretion of IL-17, IL-6, and IL-10 in DTH^+^ and cured subjects. F2 also promoted the highest frequencies of CD3^+^CD4^+^IL-2^+^TNF-α^−^IFN-γ^−^, CD3^+^CD4^+^IL-2^+^TNF-α^+^IFN-γ^−^, CD3^+^CD4^+^IL-2^+^TNF-α^−^IFN-γ^+^, and CD3^+^CD4^+^IL-2^+^TNF-α^+^IFN-γ^+^ T cells in cured and asymptomatic subjects. Consistent with this, the IFN-γ increase was correlated with decreased spleen (*R* = −0.428, *P* = 0.05) and liver sizes (*R* = −0.428, *P* = 0.05) and with increased hematocrit counts (*R* = 0.532, *P* = 0.015) in response to F1 domain, and with increased hematocrit (*R* = 0.512, *P* 0.02) and hemoglobin counts (*R* = 0.434, *P* = 0.05) in response to F2. Additionally, IL-17 increases were associated with decreased spleen and liver sizes in response to F1 (*R* = −0.595, *P* = 0.005) and F2 (*R* = −0.462, *P* = 0.04). Conversely, F1 and F3 increased the CD3^+^CD8^+^IL-2^+^TNF-α^−^IFN-γ^−^, CD3^+^CD8^+^IL-2^+^TNF-α^+^IFN-γ^−^, and CD3^+^CD8^+^IL-2^+^TNF-α^+^IFN-γ^+^ T cell frequencies of VL patients correlated with increased spleen and liver sizes and decreased hemoglobin and hematocrit values. Therefore, cure and acquired resistance to VL correlate with the CD4^+^-Th1 and Th-17 T-cell responses to F2 and F1 domains. Clinical VL outcomes, by contrast, correlate with CD8^+^ T-cell responses against F3 and F1, potentially involved in control of the early infection. The *in silico*-predicted NH36 epitopes are conserved and bind to many HL-DR and HLA and B allotypes. No human vaccine against *Leishmania* is available thus far. In this investigation, we identified the NH36 domains and epitopes that induce CD4^+^ and CD8^+^ T cell responses, which could be used to potentiate a human universal T-epitope vaccine against leishmaniasis.

## Introduction

Visceral leishmaniasis (VL) is a severe chronic vector-borne protozoan disease. Approximately 400,000 new cases of VL and 30,000 deaths are reported annually (1), and the worldwide incidence is increasing due to co-infection with HIV and the expanded geographical range of the insect vector subsequent to global warming (2). The disease is caused by *Leishmania donovani* in India, Asia, and East Africa; by *Leishmania infantum chagasi* in America; and by *Leishmania infantum* in the Middle East, Central Asia, China, and the Mediterranean (2). Bangladesh, India, Nepal, Sudan, Ethiopia, and Brazil concentrate 90% of the VL worldwide incidence (2). Clinical findings of VL range from asymptomatic cases with self-resolving infection and an anti-*Leishmania* integral immune response to severe cases characterized by intermittent fever, malaise, weight loss, cachexia, hepatomegaly, splenomegaly, hypergammaglobulinemia, anemia, leukopenia, thrombocytopenia, strong suppression of the CD4^+^ T-cell immune response, and death, if untreated (3). Chemotherapy is highly toxic, and the long-term use of this treatment can select for resistant parasites (4).

Asymptomatic subjects and cured individuals from endemic areas have an effective CD4^+^-Th1 immune response against *Leishmania* and are resistant to the disease on the basis of a positive *Leishmania*-specific delayed-type hypersensitivity (DTH^+^) skin test response (4–6). Delayed-type of hypersensitivity response (DTH) is mediated by the Th1 subset of CD4^+^ cells (7) and lost during sever VL (4–6). Identification of the antigens and HLA-restricted epitopes correlating with the natural resistance and cure of VL, and of the epitopes recognized during the severe disease is necessary to guide the development of a rational vaccine and to understand the precise immune mechanisms required for controlling parasite growth (4, 5).

Visceral leishmaniasis is associated with polarization to a Th2 immune response with increased production of IL-10 and TGF-β (8) and depletion of the Th1 cytokines IFN-γ, TNF-α, IL-2, and IL-12 produced by PBMCs in response to leishmanial lysates (4, 8, 9). The development of successful protection or resistance to VL requires, the generation of potent and durable Th1 parasite-specific memory responses, characterized by the production of IFN-γ, IL-2, and TNF-α by multifunctional CD4^+^ T cells (4, 10–13). Additionally, the assessment of the balance between immunoregulatory mechanisms, including pro-inflammatory IFN-γ and TNF-α, and the secretion of IL-17 and the regulatory cytokine IL-10 (4, 8) is required. Furthermore, CD8 T cells were also recently described, as contributing to the cure or pathology of VL (8, 14).

Many *Leishmania* antigens have been tried as potential vaccine candidates with varied immune responses and diverse species-specific protection (14–16). Leishmune^®^ is the first licensed second-generation vaccine against VL. It is composed of the FML glycoproteic antigen of *L. donovani* and saponin (17–19). Its recent use has already resulted in the reduction of the incidence of dog and human VL in Brazil (20). The nucleoside hydrolase of *L. donovani* (NH36) is the Leishmune^®^ main antigen and one of the promising candidates for vaccination against VL (17). Notably, NH36 is a vital enzyme of *Leishmania* that releases purines or pyrimidines from foreign DNA to be used in the synthesis of parasite DNA. Because it is absent from mammalian cells, it is a good target for differential chemotherapy (21, 22).

Vaccination with recombinant NH36 protein or DNA, protected mice from *L. donovani* (23), *Leishmania major* (24), *L. infantum chagasi* (25, 26), *L. mexicana* (25), and *Leishmania amazonensis* (12, 13) infections and also protected dogs infected with *L. infantum chagasi* through a Th1 immune response mediated by IFN-γ-producing CD4^+^ T cells (27). After vaccination with the recombinant NH36 in the mouse model, we described the achievement of 88% prophylactic protection (26) and 91% cure of VL (28), and 65–81% cure of cutaneous leishmaniasis (CL) (13).

NH36 is a strong phylogenetic marker of the *Leishmania* genus (11, 13, 29) that mediates high levels of vaccine cross-protection. In fact, the amino acid sequence of *L. donovani* NH36 shows high identity with the NH sequences of *L. major* (95–96%) (11, 30), *L. mexicana* (93%), *L. infantum chagasi* (99%), *L. infantum* (99%), *Leishmania tropica* (97%), *Leishmania braziliensis* (84%) (31), and *L. amazonensis* (93%) (12).

Our objective was to determine the major epitopes that contribute to protective responses, and this was done directly from the whole NH36 molecule. In fact, the *in silico* prediction disclosed the epitopes of NH36 for mice (12, 26) and human histocompatibility complex molecules (this investigation). However, although this information would allow the direct design of a synthetic epitope vaccine, the results of the immunological assays *in vivo* not always confirm the *in silico* predictions (32), and the synthetic epitopes alone are not enough immunogenic to be used as vaccine candidate antigens (14). Our strategy then was to identify through immunological assays the presence of the important epitopes in shorter sequences of the NH36 antigen that would be, therefore, more potent than the whole cognate protein and more immunogenic than the isolated epitopes.

Knowing that NH36 was a vital parasite enzyme (33, 34), a conserved molecule of the *Leishmania* genus (29), and an important antigen (35), we designed three subunit vaccines that would cover the whole sequence of NH36. Since NH36 has 314 amino acids, we subcloned and obtained its N-terminal (F1) domain (amino acids 1–103), the central (F2) domain (amino acids 104–198), and the C-terminal (F3) domain (amino acids 199–314) (26). We first vaccinated mice with NH36 and used these domains to stimulate the splenocyte secretion of IFN-γ and TNF-α (36). Furthermore, we vaccinated mice with each one of the three domains and studied the cytokine secretion and intracellular staining in response to NH36, the DTH, and the reduction of parasite load after infection with *L. infantum chagasi* (26). In both studies, F1 and F3 domains induced the strongest immune response (26, 36). However, mouse protection against *L. infantum chagasi* challenge was mediated by a CD4^+^ Th1 response directed only against F3 and was higher (88%) than that generated by the NH36 protein (68%) (26). The increases in DTH and in ratios of TNF-α/IL-10 CD4^+^-producing cells were the strong correlates of this protection, which was confirmed by *in vivo* depletion with monoclonal antibodies (26). In agreement, the *in silico* prediction identified three MHC class II-restricted epitopes in the F3 domain (26). On the other hand, prevention (12) and cure (13) of *L. amazonensis* infection in mice were determined also by a CD4^+^ T-cell-driven response to F3 but with an additional CD8^+^ T-cell response directed to the F1 domain. Coincidentally, one highly scored epitope for MHC class I-restricted molecules was detected in the sequence of F1 (12, 26). In the meantime, the predictions of one MHC class II- and two MHC class I-restricted epitopes in the F2 domain were not confirmed by any immunologic or parasitological assay developed in the mice models of VL or CL (12, 13, 26). Our results then confirmed that both, the *in silico* predictions and the *in vivo* immunological assays, are needed to improve the definition of a T-cell epitope vaccine (32).

To this point, however, the NH36 epitopes recognized by the human major histocompatibility class I and II complexes (HLAs) were not yet described. In this investigation, we aimed to identify the main domains and epitopes of NH36 to be included in a future vaccine against human VL. For that purpose, we evaluated the clinical outcomes of VL patients, cured subjects, and asymptomatic individuals of a Brazilian endemic area and assessed their correlations with cytokine expression, and with the induction of CD3^+^CD4^+^ and CD3^+^CD8^+^ multifunctional T cells, in response to the defined domains of NH36. We used these correlations as tools for identification of the domains of NH36 responsible for the cellular immune responses generated during resistance and progression of the disease. We were able to demonstrate the generation of Th1 and Th17 cells and regulatory cytokines, as well as a CD3^+^CD4^+^ Th1 multifunctional T-cell response, in cured and asymptomatic subjects. We additionally disclosed the generation of a CD3^+^CD8^+^ multifunctional T-cell response in VL patients. Finally, we identified the most immunodominant epitopes of *L. donovani* NH36 for the generation of CD4 and CD8 T cell immune responses in individuals infected with *L. infantum chagasi*. A rationale combination of the domains or epitopes that enhance both arms of T-cell immunity will contribute to the development of a universal protective or therapeutic vaccine against human VL and to the understanding of VL pathology.

## Materials and Methods

### Ethics

The protocols were performed according to the guidelines and regulations of the Brazilian National Council of Health resolution 196/96 (CAAE 0162.0.107.000-09). The protocols were performed according to the guidelines and regulations of the Brazilian National Council of Health resolution 196/96 (CAAE 0162.0.107.000-09) and were approved by the Research Ethics Committee of the Universidade Federal de Sergipe. The objectives of the study were explained to all invited participants who gave written informed consent in accordance to the Declaration of Helsinki. Participants were explained about the low risks of the procedures. Only small samples of venous blood were collected from them, and no invasive procedure was performed.

### Patients

This study was performed with patients who were admitted to the UFS University Hospital, SE, Brazil, between March 2013 and March 2015. They were clinically diagnosed with VL based on fever, weight loss, anemia, spleen and liver enlargement, pancytopenia, hypergammaglobulinemy, positive culture in NNN media (Sigma-Aldrich), and positive serum reactivity to the rK39 antigen (KalazarDetect^®^ Rapid Test, INBIOS International Inc., Seattle, WA, USA). Pregnant women, patients receiving immunosuppressive treatments, and those with diabetes or HIV or HTLV-1 co-infections were excluded. Cure was monitored 180 days after therapy with Glucantime^®^. Household contacts or patient relatives with no signs of disease were recruited and skin-tested for DTH with *Leishmania* promastigote lysate kindly provided by Centro de Produção e Pesquisa de Imunobiológicos (CPPI, Paraná, Brazil). Indurations of diameters ≥5 mm, detectable at 48 h after antigen injection, were considered positive. Hematological, hematocrit, and hemoglobin evaluation of patients and DTH^+^ subjects was performed. Increases in the spleen and liver sizes were measured in centimeters, below the ribs’ lower edge. Healthy subjects from the endemic area were included as negative controls. A total of 67 individuals were included in this study: 16 healthy controls, 14 patients with active VL, 17 cured patients, and 20 asymptomatic DTH^+^ subjects. The group of untreated patients was increased to 41 individuals in order to establish the correlations between the increases of spleen and liver sizes.

### Recombinant Antigens and Epitopes

NH36 [EMBL, *Genbank*, and DDJB databases, access number AY007193 GENBANK (AY007193 and AAG02281.1 access codes) and in SWISS-PROT (Q8WQX2 UNi-Prot access code)] and its N-terminal (F1, amino acids 1–103), central (F2, amino acids 104–198), and C-terminal (F3, amino acids 199–314) domains were cloned in *E. coli* (26), expressed, and purified as modified from the methods of Rodrigues et al. (37) and Saini et al. (38). Briefly, protein expression was induced in bacterial suspensions with 1 mM IPTG, for 4 h at 37°C. The cells were sonicated, and the insoluble pellets were washed twice with 10 mM Tris–HCl pH 8.0 and 0.5% CHAPS and further treated for 2 h at 37°C, under agitation, with a solubilization buffer composed of 20 mM Tris–HCl pH 8.0, 500 mM NaCl, 10% glycerol, and 8 M urea. Then, the suspension was homogenized by successive passages through 20-ml syringes with 1.2 mm × 40 mm needles followed by centrifugation, for 30 min at 14,000 rpm. The supernatant was loaded on a Ni-NTA chromatography column previously equilibrated with solubilization buffer. After sample application, the column was washed with three volumes of solubilization buffer containing 20 mM imidazole. Next, the column was washed with three volumes of the same buffer containing 5 mM reduced glutathione, 0.1% Triton X-100, and each one of the decreasing concentrations of a urea gradient (6–1 M), and buffer with no urea added for refolding. Elution of the proteins was achieved using 250 mM imidazole in refolding buffer without urea and confirmed by protein assay and 15% SDS-PAGE. The proteins were finally dialyzed against 50 mM Tris–HCl, pH 8, 50 mM NaCl, 50% glycerol, and 0.1 mM DTT and the absence of LPS was confirmed using the LAL QCL-1000 kit (Lonza). The purification process yielded 1 mg protein antigen per liter of bacterial culture. The homology between the sequence of *L. donovani* NH36 (GenBank: AAG02281.1) and *Leishmania infantum chagasi* NH (Lch-NH) (GenBank: AAS48353.1) was determined using NIH-NCBI Standard Protein BLAST software. A molecular model was obtained by homology modeling using the Modeller 9.10 software and data for the nucleoside hydrolase from a *L. major* template (RCSB PDB code: 1EZR; crystal structure of nucleoside hydrolase of *L. major*) (39).

For control purposes, in order to further demonstrate that NH36 is a component of SLA, we also assayed if sera of mice vaccinated with three doses of 100 μg of either NH36, F1, F2, or F3 recombinant antigens formulated with 100 μg saponin, with a weekly interval, recognized the SLA of *L. infantum chagasi* (2 μg/well) in a standard ELISA assay using peroxidase-conjugated protein A (26).

HLA-DR-binding CD4 epitopes were mapped with the TEPITOPE program. CD8 epitopes were identified using SYFPEITHI software. The predicted epitopes were synthetized by GenScript (NJ, USA). The analysis of the identity of the epitopes in the different leishmanial species was performed using the sequences of nucleoside hydrolase of *Leishmania* species of PubMed Protein Databank and the sequence of *L. amazonensis* NH A34480 (12).

### Cytokine Secretion

PBMCs were obtained from heparinized vein blood using a standard Ficoll-Hypaque procedure, washed twice with RPMI 1640 and counted microscopically with Trypan Blue. The cells were plated (2 × 10^5^/well) and stimulated with 10 μg/ml of NH36, F1, F2, and F3, stationary phase *L. donovani* promastigote lysate or with no addition for 72 h at 30°C and 5% CO_2_. The secretion of IFN-γ, TNF-α, IL-1β, IL-4, IL-6, IL-12p70, IL-10, and IL-17 into the supernatants was evaluated with a Multiplex^®^ MAP-Luminex MAP^®^ kit and analyzed using Milliplex Analist 5.1 software (Merck Millipore, Billerica, MA, USA), according to the manufacturer’s instructions. The sensitivity of the assay was established with a range of 8–15,000 pg/ml recombinant cytokines. We further assessed the IFN-γ secretion by PBMC of asymptomatic subjects in response to the synthetic predicted epitopes using the Invitrogen™ NOVEX™ IFN-γ Human Ultrasensitive Magnetic Bead kit (USA).

### Intracellular Cytokine Staining

PBMCs (2 × 10^6^/well) were *in vitro* cultured in 96-well/plates with 10 μg/ml of NH36, F1, F2, and F3, stationary phase *L. donovani* promastigote lysate or with no addition, for 6 h, followed by the addition of Brefeldin A (GolgiPlus, BD Biosciences, Franklin Lakes, NJ, USA), and further incubation for 12 h. The plates were centrifuged at 1,430 rpm for 5 min at 4°C, washed with PBS, and blocked with 2% fetal goat and 2% fetal bovine calf sera. The cells were further stained for surface markers with V500 mouse anti-human CD3 clone UCHT1 (RUO), FITC mouse anti-human CD4 clone RPA-T4 (RUO), and PE-Cy5 mouse anti-human CD8 clone RPA-T8 (RUO) monoclonal antibodies (BD Biosciences Pharmingen, San Diego, CA, USA), washed with PBS, and fixed and permeabilized with the Cytofix/Cytoperm mixture (BD Biosciences, Pharmingen, San Diego, CA, USA) for 20 min. The cells were then stained for the intracellular expression of cytokines with anti-IL-2-BV421 (clone 5344.111), anti-TNF-α-PE (clone Mab 11), and anti-IFN-γ-PE-Cy7 (clone B27) antibodies (BD Biosciences Pharmingen), washed with Perm Wash buffer (BD Biosciences Pharmingen), and resuspended in PBS. A minimum of 30,000 events were acquired on a BD FACSCanto II™ flow cytometer and analyzed using FlowJo software (Tree Star Inc., Ashland, OR, USA). All T cell frequencies were recorded after background subtraction of cells incubated without antigen.

For multiparameter cytometry analysis, the gated single-cell lymphocyte population was additionally gated for CD3 expression and subsequently for CD4 or CD8 expression. Production of each cytokine (IL-2, TNF-α, and IFN-γ) were analyzed individually inside CD3^+^CD4^+^ or CD3^+^CD8^+^ T cells gate. Boolean gating was used to generate combinations of cytokine expression and types of lymphocytes in order to identify lymphocytes expressing one cytokine or any combination of two cytokines or three cytokines (Figure S5 in Supplementary Material).

### Statistical Analysis

Kruskal–Wallis and Mann–Whitney tests were used for means comparison, and Spearman’s two-tailed correlation test was used for correlation analysis using GraphPad Prism 6.03 software. All experiments were performed at least twice, and the indicated error bars are based on the SEM.

## Results

### Clinical Outcomes of VL Patients

Clinical examination and laboratory exams of patients before treatment revealed the typical sign of VL: hepatomegaly, splenomegaly, leukopenia, neutropenia, eosinopenia, lymphopenia, monocytopenia, thrombocytopenia, and decreased hematocrit and hemoglobin levels (Table 1). Conversely and as expected for their natural resistance status to infection, all outcomes remained at normal levels in DTH^+^ asymptomatic subjects and in cured subjects, except for eosinophils, monocytes, and platelets.

**Table 1:** **Comparison of clinical outcomes**.

Clinical outcomes	VL patients	Cured patients		Asymptomatic DTH^+^ subjects	
	Mean (SE)	Mean (SE)	*P* value	Mean (SE)	*P* value
Spleen increase in cm	1.7 (0.6)	0 (0)	0.002	0 (0)	0.001
Liver increase in cm	0.8 (0.3)	0 (0)	0.045	0 (0)	0.027
Leukocytes per mm^3^	2,384 (477)	6,047 (1,234)	0.018	7,556 (674)	0.001
Neutrophils per mm^3^	712 (285)	3,407 (780)	0.010	4,941 (701)	0.001
Eosinophils per mm^3^	18.5 (7.7)	93.9 (52)	0.183	337.3 (66)	0.001
Lymphocytes per mm^3^	1,094 (244)	2,068 (368)	0.045	2,724 (454)	0.008
Monocytes per mm^3^	297 (72.3)	433 (32.4)	0.097	543 (59)	0.019
Hematocrit (%)	28.4 (2.6)	40.9 (2.3)	0.006	41.4 (1.5)	0.004
Hemoglobin (gr/dl)	8.9 (0.8)	13.7 (0.8)	0.002	13 (0.5)	0.002
Platelets per 10^−3^/μl	123.4 (25.9)	169.8 (14.4)	0.184	262.1 (14.2)	0.001

*P values express the significance of the differences to the clinical outcomes of visceral leishmaniasis (VL) patients before treatment. Means + SE. *N* = 9 VL patients before treatment, *N* = 8 cured VL patients, and *N* = 10 asymptomatic DTH^+^ subjects*.

### Expression and Purification of NH36 and Its Domains

Each bacterial clone codifying for the sequences of the recombinant NH36 and its F1, F2, and F3 domains was cultured into 2 l of bacterial culture media and induced for expression with IPTG. Conditions of expression and purification of the antigens are summarized in Figure S1A in Supplementary Material. The yield of each expression batch was 4.62 mg for NH36, 5.00 mg for F1, 3.5 mg for F2, and 3.75 mg for F3. The antigens were maintained at −80°C until use. A standardization study proved that the protein concentration was preserved at −80°C until at least 24 months after purification. SDS-PAGE analysis disclosed that the expressed proteins showed their expected molecular weights: 34,238.6 Da for NH36, 10,845.5 Da for F1, 10,327.9 Da for F2, and 13,101.1 Da for F3 (Figure S1B in Supplementary Material).

### Cytokine Secretion in Response to NH36 Domains

**Figure 1:**
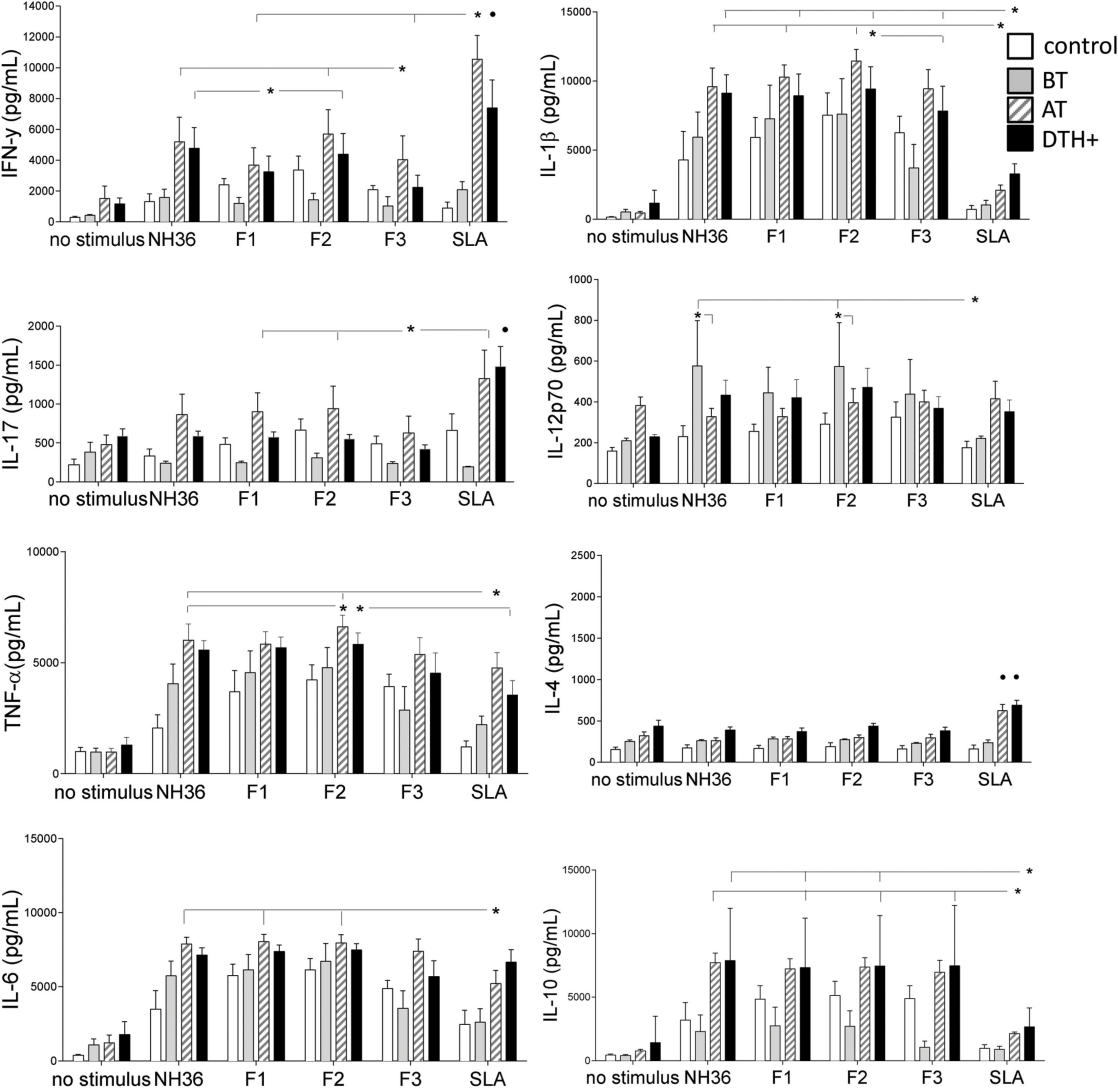
**Cytokine release in the supernatants of PBMCs after *in vitro* incubation with NH36, F1, F2, or F3**. PBMCs were isolated from venous blood and incubated *in vitro* with 10 μg/ml recombinant NH36, F1, F2, and F3 antigens, with *Leishmania donovani* stationary phase lysate, or with no addition, for 72 h. Cytokine secretion in supernatants was measured with a Multiplex^®^ MAP-Luminex assay. Asterisks and horizontal lines indicate significant differences between groups. • indicates differences in the same group of patients induced by other antigens. The means + SE are shown. *N* = 6 in the normal healthy control group; *N* = 7 in the active VL group; *N* = 9 in the cured group; and *N* = 10 in the asymptomatic DTH^+^ group.

We investigated which NH36 domains target the cellular immune response. The secretion of most cytokines was enhanced in cured and DTH^+^ subjects above the levels detected in patients before treatment, except for IL12p70 (Figure 1). Additionally, the secretion of IFN-γ, IL-17, and IL-10 was lower in patients before treatment than in healthy controls of endemic areas.

We observed discrete, but significant, differences between the immunogenicity of NH36 domains. The F2 peptide alone induced the highest levels of IFN-γ, IL-1β, and TNF-α. Furthermore, F1 and F2 domains together promoted the strongest secretion of IL-17, IL-6, and IL-10 in DTH^+^ and cured subjects (Figure 1). Noteworthy, SLA induced lower levels of IL-1β and IL-10 than F1 and F2 domains (Figure 1). F2 was also the predominant domain that secreted higher levels of IL12p70 than SLA, but in patients before treatment. Conversely, IL-4 secretion was promoted only by SLA in cured and DTH^+^ individuals (Figure 1). We also show that serum antibodies of mice vaccinated with NH36, F1, F2, or F3 domains and saponin recognize the SLA antigen of *L. infantum chagasi* in an ELISA assay, indicating that NH36 is an antigenic component of SLA (Figure S2 in Supplementary Material).

We further investigated if this Th1 response correlates with the natural resistance to VL. In fact, the IFN-γ increase in response to F1, and the secretion of IL-17 in response to F1 and F2, strongly correlates with the decreases of spleen and liver sizes, which are signals of resistance to *Leishmania* infection (Table 2; Figure S3 in Supplementary Material). The increases in liver and spleen sizes are highly correlated (*R* = 0.796, *P* < 0.0001) (Figure S4 in Supplementary Material).

**Table 2:** **Correlation between clinical status, cytokine secretion and frequencies of CD4^+^ and CD8^+^ lymphocytes secreting one, two, or three cytokines, in response to NH36 domains**.

Cytokines		NH36		F1		F2		F3	
		*R* value	*P* value	*R* value	*P* value	*R* value	*P* value	*R* value	*P* value
IFN-γ	Spleen size			−0.428	0.050				
	Liver size			−0.428	0.050				
	Hematocrit			0.532	0.015	0.512	0.020		
	Hemoglobin					0.434	0.050		

IL-17	Spleen size	−0.546	0.012	−0.595	0.005	−0.462	0.040		
	Liver size	−0.546	0.012	−0.595	0.005	−0.462	0.040		
	Monocytes	0.539	0.014	0.580	0.007				
	Hemoglobin			0.466	0.038				

IL-6	Spleen size	−0.464	0.039						
	Liver size	−0.464	0.039						

CD3^+^CD4^+^IL-2^−^TNF-α^+^IFN-γ^−^	Neutrophils	0.452	0.027	0.437	0.003				

CD3^+^CD4^+^IL-2^−^TNF-α^−^IFN-γ^+^	Monocytes			0.458	0.024				
	Hematocrit							−0.432	0.035
	Neutrophils					0.432	0.035		

CD3^+^CD4^+^IL-2^+^TNF-α^−^IFN-γ^+^	Platelets	0.476	0.019			0.454	0.026		

CD3^+^CD4^+^IL-2^+^TNF-α^+^IFN-γ^+^	Leukocytes			0.405	0.050				

CD3^+^CD8^+^IL-2^+^TNF-α^−^IFN-γ^−^	Spleen size			0.424	0.034				
	Hemoglobin			−0.534	0.006				
	Hematocrit			−0.526	0.007				

CD3^+^CD8^+^IL-2^−^TNF-α^−^IFN-γ^+^	Eosinophils			0.412	0.045				
	Monocytes	0.672	0.001	0.766	0.001	0.406	0.049	0.480	0.017

CD3^+^CD8^+^IL-2^+^TNF-α^−^IFN-γ^+^	Liver size							0.557	0.005

CD3^+^CD8^+^IL-2^−^TNF-α^+^IFN-γ^+^	Monocytes	0.566	0.004			0.599	0.002		

CD3^+^CD8^+^IL-2^+^TNF-α^+^IFN-γ^+^	Spleen size			0.445	0.029			0.403	0.051
	Liver size			0.603	0.002			0.621	0.001
	Leukocytes					0.421	0.040		
	Neutrophils					0.412	0.046		

*Non-parametric Spearman two-tailed correlation analysis between the outcomes of clinical variables and cytokines secreted in vitro, or frequencies of CD3^+^CD4^+^ and CD3^+^CD8^+^ T cells secreting 1+, 2+, and 3+ cytokines in vitro, in response to incubation with NH36, F1, F2, or F3 antigens*.

Furthermore, the increase of IFN-γ secretion in response to F1 and F2 domains correlates with the increase of hematocrit and, in response to the F2 peptide, also correlates with the increment in Hg concentration (Table 2; Figure S4 in Supplementary Material). Likewise, IL-17 secretion in response to F1 correlated with the increase in monocyte counts and Hg concentration (Table 2; Figure S4 in Supplementary Material). Increases in hematocrit, hemoglobin concentration and monocyte counts are markers of resistance and cure of VL.

We found also significant correlations between the IL-17 and IL-6 secretions promoted by NH36 and the decreases in spleen and liver weights (Table 2). Also, the increases in monocyte counts and IL-6 secretion in response to NH36 were correlated.

### Intracellular Expression of Cytokines in Response to NH36 Domains

**Figure 2:**
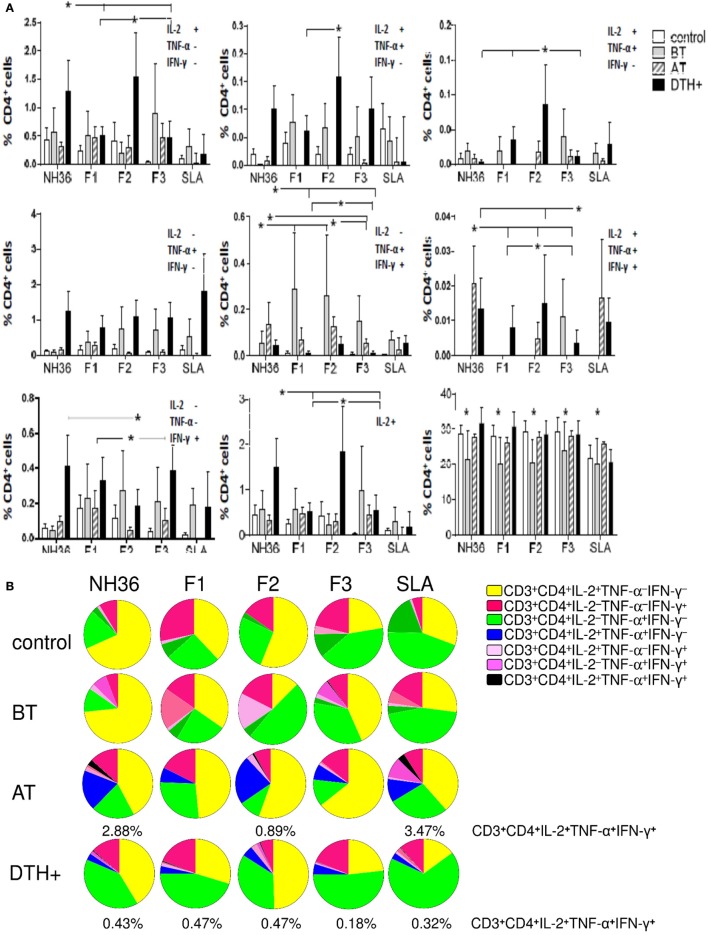
**Distinct quality of CD4 T cell response induced by NH36 or its domains**. PBMCs were incubated *in vitro* with 10 μg/ml recombinant NH36, F1, F2, and F3 antigens or with no antigenic stimulation for 6 h and further treated with brefeldin A for additional 12 h. Then, the cells were stained for surface CD3 and CD4 markers, fixed, permeabilized, and stained for the intracellular expression of IL-2, TNF-α, and IFN-γ. Multiparametric flow cytometry was used to determine **(A)** the frequencies of CD3^+^CD4^+^ lymphocytes and of CD3^+^CD4^+^ lymphocytes single producers of each one of the three cytokines (CD3^+^CD4^+^IL-2^+^TNF-α^–^IFN-γ^–^, CD3^+^CD4^+^IL-2^–^TNF-α^+^IFN-γ^–^, and CD3^+^CD4^+^IL-2^–^TNF-α^–^IFN-γ^+^), double producers (CD3^+^CD4^+^IL-2^+^TNF-α^+^IFN-γ^–^, CD3^+^CD4^+^IL-2^–^TNF-α^+^IFN-γ^+^, and CD3^+^CD4^+^IL-2^+^TNF-α^–^IFN-γ^+^), and multifunctional CD3^+^CD4^+^ T cells (CD3^+^CD4^+^IL-2^+^TNF-α^+^IFN-γ^+^); as well as the total frequencies of CD3^+^CD4^+^ cells producing IL-2 (CD3^+^CD4^+^IL2^+^) and **(B)** the fraction of the total CD3^+^CD4^+^ T cell response comprising cells expressing all three cytokines, any two cytokines, or any one cytokine in healthy individuals (control *N* = 10), active visceral leishmaniasis (VL) patients before therapy (BT *N* = 7), cured VL patients after therapy (AT *N* = 9), and in asymptomatic DTH^+^ individuals (DTH^+^ *N* = 10). The frequencies of each cytokine expressing phenotype were recorded after background subtraction of cells incubated without antigen. Results in panel **(A)** are expressed as means + SE. Asterisks and horizontal lines indicate significant differences from all other groups.

We further investigated the cellular immune response to NH36 domains by multiparameter cytometry analysis. The strategy that we used for the analysis of multifunctional T cell response using multiparameter flow cytometry is summarized in Figure S5 in Supplementary Material. The total frequencies of CD3^+^CD4^+^ T cells were reduced in patients with active VL (*P* < 0.031), compared to DTH^+^ subjects (Figure 2A). This is in agreement to the lower IFN-γ secretion found in VL patients (Figure 1).

In DTH^+^ subjects, the NH36 domains induced higher frequencies of CD4 T cells secreting one, two (except for CD3^+^CD4^+^IL-2^−^TNF-α^+^IFN-γ^+^), and three cytokines compared to those in cured individuals (*P* < 0.031) or controls (*P* < 0.031), suggesting the involvement of CD4 epitopes in natural resistance to VL (Figure 2A). Confirming the results of cytokine expression, F2 was the predominant inducer of the CD4^+^ Th1 response and alone induced the highest frequencies of CD3^+^CD4^+^IL-2^+^TNF-α^+^IFN-γ^−^ and CD3^+^CD4^+^IL-2^+^TNF-α^−^IFN-γ^+^ T cells. Furthermore, together with NH36, F2 induced the highest single (CD3^+^CD4^+^IL-2^+^TNF-α^−^IFN-γ^−^) and total (CD3^+^CD4^+^IL-2^+^) frequencies of IL-2^+^ and of multifunctional CD4 T cells (CD3^+^CD4^+^IL-2^+^TNF-α^+^IFN-γ^+^) (Figure 2A), and the lowest proportions of CD3^+^CD4^+^IL-2^−^TNF-α^−^IFN-γ^+^ T cells, in DTH^+^ and cured subjects.

Additionally, single producers of IL-2 (CD3^+^CD4^+^IL-2^+^TNF-α^−^IFN-γ^−^) represented the predominant fraction of the CD4 response in cured patients, while single producers of TNF-α (CD3^+^CD4^+^IL-2^−^TNF-α^+^IFN-γ^−^) were the major fraction in DTH^+^ subjects (Figure 2B). The F2 domain generated the most potent response, and this was confirmed by the finding that it stimulated the highest percentages of CD3^+^CD4^+^IL-2^+^TNF-α^−^IFN-γ^−^ single producers (55 and 50%), CD3^+^CD4^+^IL-2^+^TNF-α^+^IFN-γ^−^ (23 and 5%), CD3^+^CD4^+^IL-2^+^TNF-α^−^IFN-γ^+^ (3 and 3%), and CD3^+^CD4^+^IL-2^+^TNF-α^+^IFN-γ^+^ multifunctional T cells (0.89 and 47%) in cured and DTH^+^ individuals, respectively. These results confirm that this domain generates the most advanced stage of differentiation of the CD4 response (Figures 2A,B). F1 and F3, on the other hand, induced the highest frequencies of CD3^+^CD4^+^IL-2^−^TNF-α^+^IFN-γ^−^ and CD3^+^CD4^+^IL-2^−^TNF-α^−^IFN-γ^+^ single producers (Figure 2B).

We found interesting correlations between the clinical outcomes of VL and T cell immunity to NH36 antigens. As correlates of VL resistance or cure, the increases of frequencies of CD3^+^CD4^+^IL-2^−^TNF-α^+^IFN-γ^−^, CD3^+^CD4^+^IL-2^−^TNF-α^−^IFN-γ^+^, and multifunctional CD3^+^CD4^+^IL-2^+^TNF-α^+^IFN-γ^+^ T cells induced by F1 were significantly associated with the increases in neutrophil, monocyte, and leukocyte counts, respectively (Table 2). Additionally, the increases in CD3^+^CD4^+^IL-2^−^TNF-α^−^IFN-γ^+^ frequencies generated by F2 correlated with the neutrophil counts, and the increase in CD3^+^CD4^+^IL-2^+^TNF-α^−^IFN-γ^+^ correlated with the platelets counts. In contrast and as a marker of susceptibility to VL, the F3 induced CD3^+^CD4^+^IL-2^−^TNF-α^−^IFN-γ^+^ T cell frequency was inversely correlated with the hematocrit values (Table 2).

**Figure 3:**
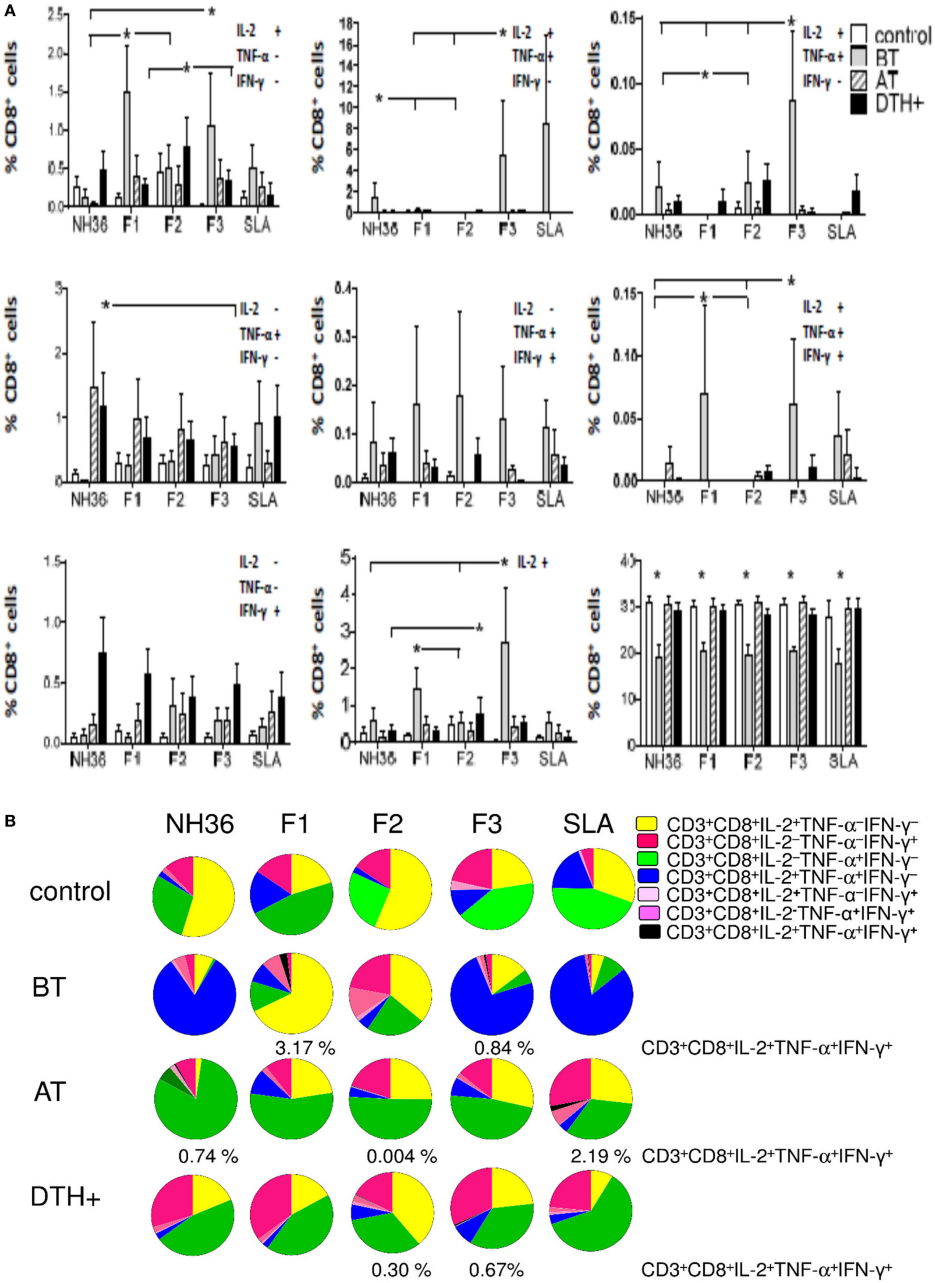
**Distinct quality of CD8 T cell response induced by NH36 or its domains**. PBMCs were incubated *in vitro* with 10 μg/ml recombinant NH36, F1, F2, and F3 antigens or with no antigenic stimulation for 6 h and further treated with brefeldin A for additional 12 h. Then the cells were stained for surface CD3 and CD8 markers, fixed, permeabilized and stained for the intracellular expression of IL-2, TNF-α, and IFN-γ. Multiparametric flow cytometry was used to determine **(A)** the frequencies of CD3^+^CD8^+^ lymphocytes, and of CD3^+^CD8^+^ lymphocytes single producers of each one of the three cytokines (CD3^+^CD8^+^IL-2^+^TNF-α^–^IFN-γ^–^, CD3^+^CD8^+^IL-2^–^TNF-α^+^IFN-γ^–^, and CD3^+^CD8^+^IL-2^–^TNF-α^–^IFN-γ^+^), double producers (CD3^+^CD8^+^IL-2^+^TNF-α^+^IFN-γ^–^, CD3^+^CD8^+^IL-2^–^TNF-α^+^IFN-γ^+^, and CD3^+^CD8^+^IL-2^+^TNF-α^–^IFN-γ^+^), and multifunctional CD8 T cells (CD3^+^CD8^+^IL-2^+^TNF-α^+^IFN-γ^+^); as well as the total frequencies of CD8 T cells producing IL-2 (CD3^+^CD8^+^IL2^+^) and **(B)** the fraction of the total CD3^+^CD8^+^ T cell response comprising cells expressing all three cytokines, any two cytokines, or any one cytokine in healthy individuals (control *N* = 10), active visceral leishmaniasis (VL) patients before therapy (BT *N* = 7), cured VL patients after therapy (AT *N* = 9), and in asymptomatic DTH^+^ individuals (DTH^+^ *N* = 10). The frequencies of each cytokine expressing phenotype were recorded after background subtraction of cells incubated without antigen. Results in panel **(A)** are expressed as means + SE. Asterisks and horizontal lines indicate significant differences from all other groups.

Additionally, the analysis of the cytotoxic response disclosed that the total frequency of CD3^+^CD8^+^ T cells (Figure 3A) was lower in patients and higher in DTH^+^ and cured subjects. However, unlike the predominant enhancement of CD3^+^CD4^+^ T-cell frequencies, observed in DTH^+^ individuals (Figure 2A), the frequencies of CD3^+^CD8^+^ T cells producing cytokines, except for the single producers of TNF-α (CD3^+^CD8^+^IL-2^−^TNF-α^+^IFN-γ^−^) and IFN-γ (CD3^+^CD8^+^IL-2^−^TNF-α^−^IFN-γ^+^), were higher in patients with active VL (*P* < 0.028–0.050) (Figure 3A), suggesting the importance of CD8 epitopes in the development of the disease.

F1 and F3 induced the highest single and total frequencies of CD8 T cells secreting IL-2 (CD3^+^CD8^+^IL-2^+^TNF-α^−^IFN-γ^−^ and CD3^+^CD8^+^IL-2^+^) and of multifunctional CD8 T cells (CD3^+^CD8^+^IL-2^+^TNF-α^+^IFN-γ^+^), and F3 alone induced the highest proportions of CD3^+^CD8^+^IL-2^+^TNF-α^+^IFN-γ^−^ and CD3^+^CD8^+^IL-2^+^TNF-α^−^IFN-γ^+^ T cells (Figure 3A). F2 promoted low frequencies of CD3^+^CD8^+^IL-2^+^TNF-α^−^IFN-γ^−^ cells in DTH^+^ subjects, NH36 induced higher frequencies of CD3^+^CD8^+^IL-2^−^TNF-α^+^IFN-γ^−^ T cells in cured patients, while the CD3^+^CD8^+^IL-2^−^TNF-α^−^IFN-γ^+^ T-cell frequencies were enhanced in DTH^+^ individuals (*P* < 0.0286), regardless of the antigen used (Figure 3A).

Furthermore, in patients before treatment, F1 and F3 induced the highest percentages of CD3^+^CD8^+^IL-2^+^TNF-α^+^IFN-γ^+^ multifunctional cells (3.17 and 0.84%, respectively), while F1 promoted the highest fraction of the CD3^+^CD8^+^IL-2^+^TNF-α^−^IFN-γ^−^ T cells (68%) and F3, the highest contribution of CD3^+^CD8^+^IL-2^+^TNF-α^+^IFN-γ^−^ T cells (74%) (Figure 3B). Cured and DTH^+^ subjects showed an enhanced fraction of CD3^+^CD8^+^IL-2^−^TNF-α^+^IFN-γ^−^ secreting lymphocytes. A multifunctional CD3^+^CD8^+^ T cell contribution was also found in cured patients, in response to F2 and in DTH^+^ subjects in response to F2 and F3. Interestingly, F1 and F3 promoted the highest frequencies of CD8 T cells secreting only IFN-γ (CD3^+^CD8^+^IL-2^−^TNF-α^−^IFN-γ^+^), in DTH^+^ subjects (Figure 3B).

In addition, we analyzed if the cytotoxic response was correlated with the clinical outcomes (Table 2). As a marker of the advancement of the disease, F1 induced increased frequencies of CD3^+^CD8^+^IL-2^+^TNF-α^−^IFN-γ^−^ T cells, which were correlated with increased spleen sizes and decreased hemoglobin and hematocrit values. Additionally, F1, together with F3, increased the frequencies of multifunctional CD3^+^CD8^+^ T cells, which were correlated with the increased spleen and liver sizes (Table 2). As an additional marker of the progression of the disease, F3 also induced an increase in the frequencies of CD3^+^CD8^+^IL-2^+^TNF-α^−^IFN-γ^+^ T cells, which correlated with an increase in liver size. Conversely, as a marker of resistance, F2 increased the frequency of multifunctional cells, which was correlated with increased leukocyte and neutrophil counts. Furthermore, F2, together with NH36, enhanced proportions of CD3^+^CD8^+^IL-2^−^TNF-α^+^IFN-γ^+^ T cells which were correlated with the monocyte counts (Table 2).

Additionally, the cytokine responses of the CD4^+^ (Figure 2A) and CD8^+^ T cells (Figure 3A) induced by the SLA antigen were inferior, or did not differ from those promoted by NH36 and its domains.

### Mapping of the CD4^+^ and CD8^+^ T Cell Epitopes

**Figure 4:**
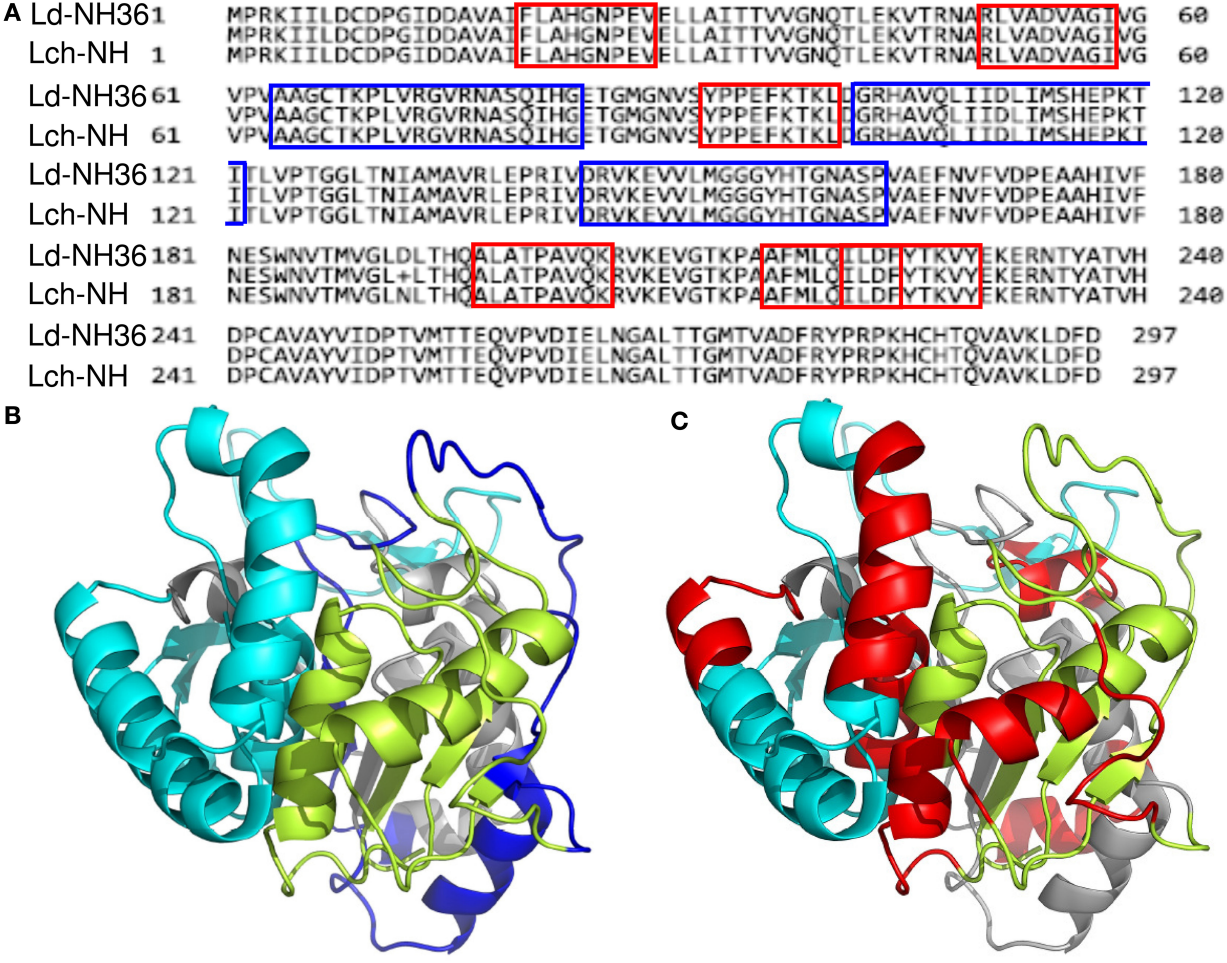
**Spatial distribution of epitopes in the monomer of the *Leishmania donovani* nucleoside hydrolase NH36 (Ld-NH36) and homology to the sequence of *Leishmania infantum chagasi* NH (Lch-NH)**. **(A)** Blast of the sequences of Ld-NH36 and Lch-NH with the sequences of the predicted epitopes for HLA class II molecules identified with a 3% threshold by the TEPITOPE program shown in blue boxes and the epitopes for HLA class I molecules identified by the SYFPEITHI software shown in red boxes. **(B)** The NH36 monomer model obtained by homology modeling to the sequence of the nucleoside hydrolase of *Leishmania major*. The model shows the N-terminus (F1, amino acids 1–103) in lime green, the central domain (F2, amino acids 104–198) in gray, and the C-terminus (F3, amino acids 199–314) in cyan. The CD4^+^ T-cell epitopes are plotted in dark blue, and **(C)** the sequences of the CD8^+^ T-cell epitopes are labeled in red.

Aiming to map the epitopes responsible for the described T cell response against NH36 domains, we first compared the sequence of the gene of *L. donovani* NH36 to the sequence of the NH gene of the close related species *L. infantum chagasi*, which is the etiological agent of VL in Brazil (Figure 4A). While NH36 of *L. donovani* is composed of 314 amino acids, the NH of *L. infantum chagasi* was described as containing 297 amino acids. The Blast analysis comparing the two sequences indicated 99% homology (Figure 4A). The difference between the two proteins is based on only one amino acid at position 192. The aspartate (D) residue of *L. donovani* NH36 is substituted with asparagine (N) in the Lch-NH sequence (Figure 4A). This result suggests that the recombinant domains of *L. donovani* NH36 could generate a successful cross-protection against infection by *L. infantum chagasi*.

Additionally, confirming our experimental results, the TEPITOPE program, with a 3% threshold, identified one epitope for CD4 T cells in the F1 domain and two epitopes in the F2 domain of NH36 (Table 3). These three CD4 epitopes showed high percent of predicted binding to HLA-DR molecules (Table 3) and are highly promiscuous. In fact, the epitope “AAGCTKPLVRGVRNASQIHG” of F1 (64–93) binds to 20 among the 25 most frequent human HLA DR molecules (DRB1*0301, DRB1*0401, DRB1*0402, DRB1*0404, DRB1*0405, DRB1*0410, DRB1*0801, DRB1*0802, DRB1*0804, DRB1*0806, DRB1*1101, DRB1*1104, DRB1*1106, DRB1*1107, DRB1*1305, DRB1*1307, DRB1*1311, DRB1*1321, DRB1*1501, and DRB1*1502). The epitope “GRHAVQLIIDLIMSHEPKTI” of F2 (102–121) binds to 21 of the 25 most frequent human HLA DR molecules (DRB1*0102, DRB1*0301, DRB1*0401, DRB1*0402, DRB1*0404, DRB1*0405, DRB1* 410, DRB1*0421, DRB1*0801, DRB1*0802, DRB1*0804, DRB1*0806, DRB1*1101, DRB1*1104, DRB1*1106, DRB1*1107, DRB1*1305, DRB1*1311, DRB1*1321, DRB1*1501, and DRB1*1502). Additionally, the epitope “DRVKEVVLMGGGYHTGNASP” of F2 (144–163) binds to 19 of the 25 most frequent human HLA DR molecules (DRB1*0101, DRB1*0102, DRB1*0404, DRB1*0405, DRB1*0410, DRB1*0801, DRB1*0802, DRB1*0804, DRB1*0806, DRB1*1101, DRB1*1104, DRB1*1106, DRB1*1107, DRB1*1307, DRB1*1311, DRB1*1321, DRB1*1501, DRB1*1502, and DRB5*0101) (Figures 4A,B).

**Table 3:** **Peptide sequences of the NH36 antigen selected by the TEPITOPE and SYFPEITHI algorithms**.

Amino acid location	Lymphocytes	Domain	Sequences	Prediction of molecular binding	
				HLA-DR (%)^a^	HLA-A and B scores^b^
64–93	CD4	F1	AAGCTKPLVRGVRNASQIHG	80	–
102–121	CD4	F2	GRHAVQLIIDLIMSHEPKTI	84	–
144–163	CD4	F2	DRVKEVVLMGGGYHTGNASP	76	–
20–28	CD8	F1	FLAHGNPEV	–	28 (A*02:01)
92–100	CD8	F1	YPPEFKTKL	–	21 (B*0702)
197–205	CD8	F3	ALATPAVQK	–	34 (A*03)
211–219	CD8	F3	AFMLQILDF	–	19 (B*4402) 17 (A*2402)
221–229	CD8	F3	ILDFYTKVY	–	27 (A*01)

*^a^Predictions were calculated using the TEPITOPE algorithm using 3% threshold*.

*^b^Predictions obtained using the SYFPEITHI algorithm*.

Also supporting our experimental results, two and three CD8 epitopes for the HLA class I molecules were predicted in the sequences of F1 and F3 domains, respectively (Table 3), by the SYFPEITHI software (Figures 4A,C), while no epitope for CD8 was predicted in the sequence of F2.

The CD4 and CD8 epitopes (Figure 4A) are completely conserved and have identical composition in the sequences of NH of *L. donovani, L. infantum chagasi*, and *L. infantum* (Tables 4 and 5), which are the three species causing VL. Minor variations in 1–3 or 1 amino acids were detected in the CD4^+^ and CD8^+^ epitopes, respectively, of all other species, which also belong to the subgenus *Leishmania*. Additionally, variations in 3–5 and 1–4 amino acids were observed in the sequences of CD4^+^ and CD8^+^ epitopes of *Leishmania panamensis* and *L. braziliensis*, which are species that belong to the genus *Viannia* (Tables 4 and 5). The presence of these highly conserved epitopes explain the high degree of cross-species recognition displayed by lymphocytes of patients infected with *L. infantum chagasi* against the *L. donovani* NH36 recombinant domains.

**Table 4:** **Conserved epitopes for CD4^+^ T cells within the genus *Leishmania***.

*Leishmania* species	Epitope CD4-F1 (64–93)	Epitope CD4-F2 (102–121)	Epitope CD4-F2 (144–163)
*L. donovani*	AAGCTKPLVRGVRNASQIHG	GRHAVQLIIDLIMSHEPKTI	DRVKEVVLMGGGYHTGNASP
*L. infantum chagasi*	AAGCTKPLVRGVRNASQIHG	GRHAVQLIIDLIMSHEPKTI	DRVKEVVLMGGGYHTGNASP
*L. infantum*	AAGCTKPLVRGVRNASQIHG	GRHAVQLIIDLIMSHEPKTI	DRVKEVVLMGGGYHTGNASP
*L. amazonensis*	AAGCAKPLVRGVRNASQIHG	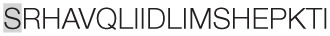	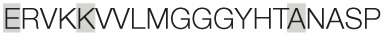
*L. major*	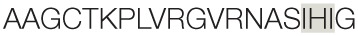	GRHAVQLIIDLIMSHEPKTI	DRVKEVVLMGGGYHTGNASP
*L. tropica*	AAGCTKPLVRGVRNASQIHG	GRHAVQLIIDLIMSHEPKTI	DRVKEVVLMGGGYHTGNASP
*L. mexicana*	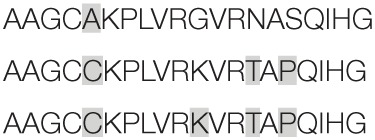
*L. braziliensis*		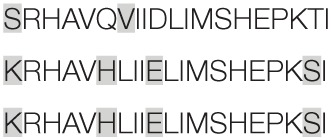
*L. panamensis*			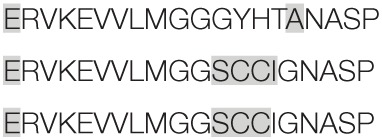

*The amino acids that are different from those of the sequence described in the Nucleoside hydrolase NH36 of L. donovani*.

**Table 5:** **Conserved epitopes for CD8^+^ T cells of the genus *Leishmania***.

*Leishmania* species	CD8-F1 (20–28)	CD8-F1 (92–100)	CD8-F3 (197–205)	CD8-F3 (216–224)	CD8-F3 (221–229)
*L. donovani*	FLAHGNPEV	YPPEFKTKL	ALATPAVQK	AFMLQILDF	ILDFYTKVY
*L. infantum chagasi*	FLAHGNPEV	YPPEFKTKL	ALATPAVQK	AFMLQILDF	ILDFYTKVY
*L. infantum*	FLAHGNPEV	YPPEFKTKL	ALATPAVQK	AFMLQILDF	ILDFYTKVY
*L. amazonensis*	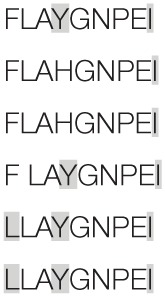	YPPEFKTKL	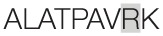	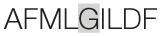	ILDFYTKVY
*L. major*		YPPEFKTKL	ALATPAVQK	AFMLQILDF	ILDFYTKVY
*L. tropica*		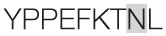	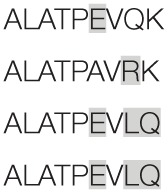	AFMLQILDF	ILDFYTKVY
*L. mexicana*		YPPEFKTKL		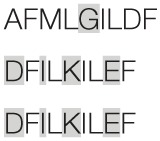	ILDFYTKVY
*L. braziliensis*		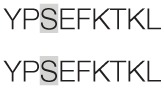			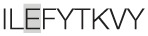
*L. panamensis*					ILEFYTEVY

*The amino acids that are different from those of the sequence described in the Nucleoside hydrolase NH36 of L. donovani*.

**Figure 5:**
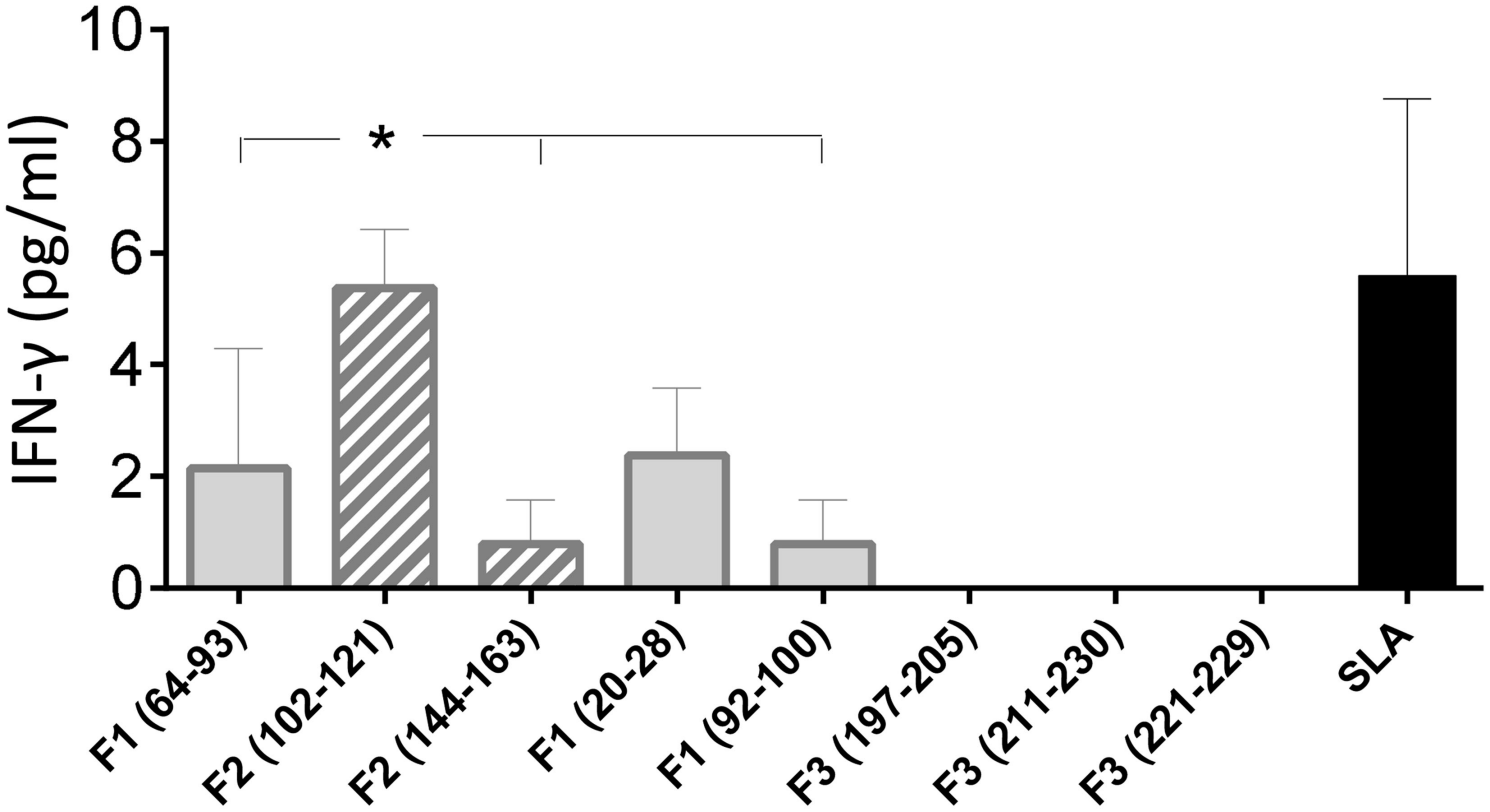
**IFN-γ secretion induced by the synthetic predicted epitopes of NH36**. PBMCs of asymptomatic subjects (*N* = 6) were incubated *in vitro* with 10 μg/ml of the synthetic predicted epitopes of NH36 or with *Leishmania donovani* stationary phase lysate, or with no addition, for 72 h. IFN-γ secretion in supernatants was measured with Invitrogen NOVAK Magnetic Beads Assay. Asterisks and horizontal lines indicate significant differences between groups. The means + SE are shown.

In order to confirm the accuracy of the *in silico* predictions, the CD4 predicted epitopes of F2 and F1 and the CD8 predicted epitopes of F1 and F3 domains were chemically synthetized and incubated *in vitro* with PBMC of asymptomatic DTH^+^ subjects. The secretion of IFN-γ to PBMC supernatants in response to those epitopes was assessed by an ELISA assay (Figure 5). The three predicted epitopes for CD4^+^ T cells promoted IFN-γ secretion. The IFN-γ response generated by the epitope F2 (102–121) was 85% higher (mean = 5.35 pg/ml; *P* = 0.006) than that induced by the F2 epitope (144–163) (mean + 0.79 pg/ml), and 60% stronger (*P* = 0.050) than that induced by the F1 (64–93) epitope (mean = 2.14 pg/ml) (Figure 5). Levels of IFN-γ secreted after incubation with the F2 (102–121) epitope were not different from those obtained in response to the SLA complex antigen indicating that this is the major epitope responsible for the immunodominance of the F2 domain detected in all previous immunological assays. IFN-γ secretion was also directed against two predicted epitopes for the CD8 T cells of the F1 domain (sequences 20–28 and 92–100), which were no different from SLA antigen, but not for the epitopes of the F3 domain (Figure 5).

## Discussion

Patients of this study showed the typical clinical outcomes of VL (3, 4, 8, 40). On the other hand, no alterations were detected in cured or DTH^+^ individuals, whose PBMCs secreted the most pro-inflammatory cytokines, mainly in response to F2 and F1, and also secreted higher levels of TNF-α and IL-1β than those induced by the *L. donovani* lysate. The antigenic predominance of F2 is related to the finding of two predicted CD4 T cell epitopes in its sequence, while only one epitope is located in F1, and none is located in F3. Confirming these results, the additional increases in the secretion of IFN-γ and IL-17, in response to F2 and F1 were highly correlated with the reduction of VL clinical signs, suggesting that F2 and F1 are indeed the NH36 markers of the Th1-mediated cure or resistance to VL (9). In agreement to those results, the epitopes (102–121 and 144–163) of F2 and the sequence (64–93) of F1 induced the highest IFN-γ responses in DTH^+^ individuals. Therefore, our immunological results supported the *in silico* predictions of epitopes for CD4^+^ T cells.

Epidemiological studies performed in Brazil (6, 41, 42) and Kenya (43) have shown that a positive DTH response is a marker for developing acquired resistance to human VL (5, 41, 43). In the north east of Brazil, where the present study was also developed, the DTH^+^ response was proved to be under genetic control (6, 42, 43) with some genotypes associated to DTH^+^ subjects and other to DTH^−^ subjects (42). A DTH positive phenotype is considered not only a measure of acquired resistance to natural infection (41, 43), but also, the only correlate with date of protection in human vaccine trials (44–46). Our results are thus relevant considering that we described the conserved domains and epitopes of *L. donovani, L. infantum*, and Lch-NHs that promote Th1 responses in DTH^+^ subjects and, because of that, which correlate with the resistance to *L. infantum chagasi* infection.

While the hallmark of VL is associated with an impairment of Th1 responses and a depressed cell-mediated immune response characterized by the failure of PBMCs of untreated patients to produce IFN-γ in response to leishmanial antigens (47–49), in our study, the F2 peptide alone promoted the highest secretion of IFN-γ, IL-1β, and TNF-α in cured and DTH^+^ subjects. In fact, the IFN-γ and TNF-α levels produced by DTH^+^ subjects in response to F2 are two or three orders of magnitude higher, respectively, than those detected in cured patients from India in response to SLA, and one order of magnitude higher than those obtained in response to the antigens LACK and TRYP (4). Also, similar to our results, cured patients of India produce IFN-γ and TNF-α in response to antigens (48, 49) providing correlates of resistance (4). Additionally, Th1 responses including a robust TNF-α response directed against NH36 were also recently described in human healthy subjects vaccinated with an NH36-fusion protein (11). The pro-inflammatory enhanced cytokine secretion in response to F2 and F1 domains indicated that they are the targets of the anti-NH36 immune response related to VL resistance.

We therefore revealed in our study the main domains and epitopes of NH36 which can be considered as potential candidates for a T-epitope vaccine for humans. Indeed, while prediction programs and pre-clinical studies in mice indicated the importance of three CD4 epitopes of F3, and two of F1 (26) in the generation of protection against visceral and CL, our study conversely describes two CD4 epitopes for humans in the F2 and one in F1. Therefore, the F2 domain, which is not relevant for mouse immunity (12, 13, 26), is however responsible for the generation of the strongest Th1 responses in humans.

IL-1β, which is highly induced by F2, is a pro-inflammatory cytokine secreted by monocytes and macrophages, which promotes inflammatory responses that activate protective immunity. In agreement to that, the increase in IL-1β secretion of cured and DTH^+^ subjects of the same endemic area, was associated with the decrease of the parasite load of infected macrophages (50). Furthermore, host resistance to infections by *L. major* (24), *L. amazonensis, L. braziliensis*, and *L. infantum chagasi*, which share a high degree of identity in their NH sequences (13, 31), has also been described to be mediated by IL-1β (51). Thus, the IL-1β secretion induced by the F2 domain could contribute to the basis of this cross-species resistance induced by NH36.

IL-12 secretion by VL untreated patients was higher in response to F2 than to SLA. As in our study, similar or higher levels of IL-12 were detected in the sera of infected untreated patients and subclinical DTH^+^ subjects from Bangladesh (52) and Brazil (53). IL-12 is associated with macrophage activation, parasite phagocytosis, and a protective host response that upregulates IFN-γ synthesis, cytolytic activity, and Th1 cell differentiation (3). Therefore, the finding of increased levels of IL-12p70 in untreated patients in response to F2 might indicate its involvement in the generation of an early inflammatory response as an attempt to control infection (53, 54).

Enlarged spleen and livers and decreased hemoglobin, albumin, and hematocrit counts correlate with the clinical variables (55–58) and the increased blood parasite load found in VL (55). By contrast, in our study, the increase of both IL-17 and IL-6 secretion in response to F1 and F2 correlated with decreases in spleen and liver sizes, which are parameters of cure and asymptomatic VL (56, 58).

The increased secretion of IL-17 in response to F1 also correlated with an increase in monocytes as well as with an increase hemoglobin counts. IL-17 is a classical effector of innate immunity that induces the expression of many inflammatory mediators, including IL-6 (8). Our results then indicate the efficient induction of a Th17 response by the F2 and F1 domains that contributes in the cure and control of the infection.

Besides inducing Th1 and Th17 responses, the F2 and F1 domains also promoted the secretion of IL-10, a cytokine which was correlated with immunosuppression defects (8, 59) and, together with IL-6, with the severity of human VL (60). This kind of mixed Th1/Th2 response was also previously described for VL (49). In fact, in our study, IFN-γ, IL-17, and IL-10 were co-expressed by PBMCs of healthy, cured, and DTH^+^ subjects and their secretion was lower in untreated patients. Since DTH^+^ is a marker of acquired resistance to VL (5, 42, 43), the increased IL-10 secretion in response to NH36 domains, in cured and DTH^+^ subjects is more associated with resistance and cure of VL than with a Th2 response. As we described here for the NH36 domains, another *Leishmania* vaccine candidate, the LaPSA-38S antigen induced specific Th1 responses and protection in mice and promoted significant levels of IFN-γ, granzyme B, and IL-10 in patients cured from cutaneous *L. major* infection and in high responders asymptomatic subjects infected with *L. major* or *L. infantum* (61). A higher secretion of TNF-α in response to LaPSA-38S was also found in the *L. infantum* high responders (61). As described for LaPSA-38S protein, the NH36 domains were able to induce a mixed Th1 and Th2/Treg cytokine response in individuals with immunity to *L. infantum chagasi* indicating that it may be exploited as a vaccine candidate. The IL-10 elevated secretion in response to NH36 domains might be determined by the increased secretion of TNF-α (60, 62) and might serve as a tool for control of tissue damage caused by the enhanced inflammatory response. The increased secretion of IL-10 in response to NH36 also suggests the presence of Treg epitopes in its domains. Confirming that, it was described that the parasite-induced IFN-γ and IL-1β act on DCs and macrophages to promote the production of IL-27, which blocks the generation of Th17 cells and facilitates the generation of IL-10-producing T cells (8). Remarkably, this ability of modulating the immune response, and promoting similar levels of IFN-γ, TNF-α, and IL-10 associated to resistance was also found after human vaccination with an NH36-fusion protein (11), in DTH^+^ asymptomatic CL patients (63), in *L. infantum*-infected asymptomatic dogs (64), and in mice vaccinated with F1 (26). Therefore, our results suggest that a combination of CD4 Th1 and/or potential regulatory epitopes of F2 and F1 modulate the human immune response against the parasite. This seems to be also the case for IL-6, which is produced by APCs and involved in the control of Th1/Th2 differentiation during CD4 T cell activation and is found in the plasma of VL patients and asymptomatic subjects in Brazil (65). The F2 and F1 domains could be therefore the basis of tools for prevention, control, or new methods of immunotherapy of this severe potentially lethal disease.

Optimal vaccine protection is achieved by developing a population of multifunctional IL-2^+^TNF-α^+^IFN-γ^+^ producing CD4 T cells that can mediate effector functions quickly, and by having a memory T-cell reservoir that secretes IL-2, TNF-α, or both and shows effector potential (66). Consistent with this, F2 induced the most robust cytokine responses and the highest frequencies of CD3^+^CD4^+^IL-2^+^TNF-α^+^IFN-γ^+^ effector T cells, CD3^+^CD4^+^IL-2^+^TNF-α^−^IFN-γ^−^, and CD3^+^CD4^+^IL-2^+^TNF-α^+^IFN-γ^−^ T cells in asymptomatic individuals. F2 also most efficiently promoted the expression of IL-2 and TNF-α by CD4^+^ T cells and promoted the development of multifunctional T cells in cured patients, followed by F1 in DTH^+^ subjects. The correlations observed between the increases in proportions of multifunctional, CD3^+^CD4^+^IL-2-TNF-α^+^IFN-γ^−^, and CD3^+^CD4^+^IL-2^−^TNF-α^−^IFN-γ^+^ T cells and leukocytes, neutrophils, and monocytes induced by F1 also confirm that, for some variables, F1 is co-dominant with F2. The increased IFN-γ secretion of PBMC of asymptomatic subjects in response to the predicted CD4^+^ epitopes of F2 (102–121 and 144–163) and F1 (69–93) explains those results. Confirming the relevance of NH36 in cross-species protection, enhanced frequencies of anti-NH36 IL-2, TNF-α, or IFN-γ-producing CD4^+^ T cells, indicating the generation of a memory-response, have also been reported, in asymptomatic patients of Bangladesh (11) and in mice vaccinated after *L. amazonensis* infection (12, 13, 25, 26).

Unlike CD4 lymphocytes, following antigenic stimulation, naïve CD8 T cells differentiate into activated effector cells that can transform into CD8^+^IL2^−^TNF-α^+^IFN-γ^+^ effector cells or into multifunctional CD8^+^IL2^+^TNF-α^+^IFN-γ^+^ memory cells (66). We described that the CD8^+^ T cells response against F1 and F3 is related to the advancement of the disease as most of the CD8^+^ phenotypes are increased in untreated patients. However, positive correlations were found between the increases in CD3^+^CD8^+^IL2^+^TNF-α^−^IFN-γ^−^, CD3^+^CD8^+^IL2^+^TNF-α^+^IFN-γ^+^, and CD3^+^CD8^+^IL2^+^TNF-α^−^IFN-γ^+^ T cell frequencies and the increases in spleen and liver sizes. These positive correlations indicate that, in these untreated patients, the CD8^+^ T cells could be involved in an early attempt to control the parasite loads of liver and spleens. Supporting the multiparameter analysis two synthetic predicted CD8^+^ epitopes of the F1 domain induced the IFN-γ secretion by PBMC of DTH^+^ subjects.

In agreement to our results, in CL models, CD8 T cells producing IFN-γ were considered as very important for directing Th2-type responses toward Th1 (67) and for establishing long-term memory that protects against re-infections (68). Memory CD8 T cells have been shown to be responsible for resistance against re-infection (69) and for promoting long-lasting protection, which is lost in their absence (70, 71). This evidence could also explain our finding of high percentages of CD3^+^CD8^+^IL-2^−^TNF-α^−^IFN-γ^+^ single positive cells observed in the DTH^+^ group, which represent the immune resistance to VL, after stimulation with F1 and F3 domains.

We further described the prediction of two promiscuous epitopes for CD4^+^ lymphocytes in the F2, and one in the F1 domain, which are all capable of binding to at least 19 of the 25 most frequent human HLA-DR molecules. These predictions were confirmed by the ability of F2 and F1 domains to induce CD4 Th1 responses, and the competence of the respective synthetic predicted epitopes, mainly the F2 (102–121) followed by the F1 (64–93), to promote the IFN-γ secretion by PBMC of DTH^+^ subjects. These results are very impressive considering that HLA class II prediction is more difficult and less reliable (14).

In this investigation, we demonstrated that F2 and F1 domains induce a strong CD4^+^-Th1 response in cured and DTH^+^ subjects, whereas the F1 and F3 domains promote a higher CD8^+^ T cell pro-inflammatory response in untreated patients and an increase in the CD3^+^CD8^+^IL-2^−^TNF-α^−^IFN-γ^+^ T cell proportions in DTH^+^ subjects. Both CD4 and CD8 responses can be related to the control and resistance to *L. infantum chagasi* infection.

Our objective in this study was to determine which are the more immunogenic domains of NH36. This would allow us to confirm the relevance of the epitopes disclosed by the *in silico* prediction programs and to define a rationale combination of the domains and/or epitopes to be used in a future universal vaccine against leishmaniasis, capable of enhancing both arms of T-cell immunity.

The epitopes were indeed identified by the prediction programs directly, on the sequence of the whole NH36 molecule, both for mice (26, 28) and human histocompatibility complex molecules (this manuscript). However, although the identification of the epitopes would allow the direct design of a synthetic epitope vaccine, it has been reported that the results of the immunological assays *in vivo* not always confirm the *in silico* predictions (32), and that the synthetic epitopes alone are not enough immunogenic to be used as vaccine candidate antigens (14). Our strategy then was to identify through immunological assays the presence of the important epitopes in the domains of the NH36 antigen that would therefore be more immunogenic than the whole cognate NH36 protein and more potent than the isolated epitopes, when used in vaccination.

The reason for evaluating the individual domains of the same antigen in order to identify the epitopes is that the domains, which contain potent epitopes, will be more potent than the whole antigen. This will indeed allow the selection of the truly relevant epitopes among all epitopes disclosed by the *in silico* software. The immunogenic domains in fact concentrate the immunogenic power of the whole antigen. Hence, for instance, if we use for incubation with PBMC one molecule of NH36, among its 314 amino acids, 40 of them compose the sequences of the three epitopes for CD4 located in F3. This means that one molecule of NH36 has 12.7% (40/314) of its sequence constituted by epitopes for CD4 T cells. If by contrast, we vaccinate only with a molecule of F3, which is composed by 115 amino acids, the 40 amino acids of the CD4 epitopes would represent now 34.8% (40/115) of the antigen, and this is what induce a threefold increase in the immunogenic effect. We are, in this way, exposing PBMC to a higher molar concentration of the relevant epitopes. That is why more protection is expected to be generated by the domain that contains the epitopes than by the whole NH36. A higher molar concentration of the epitopes is expected to be found in it than in the whole cognate protein.

In agreement with this idea, the F3 vaccine promoted in mice a 36% higher average protection than the NH36 vaccine. This average increased protection induced by the F3 vaccine above the level promoted by the NH36 vaccine included: a 32.06% higher IDR, 24 h after immunization, a 34.1% higher IDR 48 h after immunization, a 21.4% higher IDR 48 h after challenge, a 37.39% enhanced IFN-γ/IL-10 CD4^+^ T cell ratios, a 27. 18% stronger reduction of parasite load by *in vivo* depletion with anti-CD4 monoclonal antibody, a 57.99% increment in reduction of the parasite load of *L. chagasi*, and a 47% reduction in parasite load by *L. amazonensis* (26). That means that while the NH36 vaccine reduces the *L. chagasi* parasite load in 37%, the F3 reduces it in 88% (26).

Additionally, the F3 vaccine was 40% average more protective than the NH36 vaccine against *L. amazonensis* infection (28). This included a 27.58% stronger IDR, 48 h after challenge, a 20.04 and 11.64% enhanced secretion of IFN-γ and TNF-α to supernatants, respectively, and a 93.03% reduced *L. amazonensis* parasite load (28). The calculation was performed according the following equation = (F3 − NH36/F3) values × 100 and allowed us to obtain the protective effect increment (26).

Noteworthy, for mice, the prediction software had disclosed two epitopes for CD4 in F1, one in F2, and three in F3. On the other hand, one epitope for CD8 T cells was disclosed in F1 and two in F2 (26). The immunological *in vivo* assays, on the other hand, only confirmed the relevance of the three epitopes for CD4 of F3 and of the single epitope for CD8 T cells of F1 (26, 28, 29). Therefore, the immunological assays only partially confirmed the *in silico* prediction for the mice model (32). No response against the F2 domain was observed in vaccinated mice. In fact, the F3 domain induced the strongest CD4-mediated protection against VL infection (26) and, besides F3, the F1 domain induced an additional CD8-mediated response against *L. amazonensis* infection (26, 28).

Therefore, if the domains, which contain the important epitopes, are more potent than the whole protein, the need of evaluation the individual domains in order to confirm the epitopes is justified. In order to increase potency and optimize a vaccine, for instance, the domains could be even combined in a chimera that would be expected to be even more potent than the single domains. Our results support those of Kao et al. for the *Pseudomonas aeruginosa* Type IV pilus vaccine (72). The authors compared antisera raised against the PAK strain monomeric pilin protein (29−144) and a synthetic peptide containing the main receptor-binding domains (RBDs) of the pylus (128−144). They showed that not only does the synthetic peptide generated higher titers of RBD-specific antibodies but that the anti-peptide antibodies have a higher affinity for the native protein than the anti-pilin (72). The strategy of using the domains that concentrate the most relevant epitopes was the basis of the development of the development of the vaccines against human malaria (central and C-terminal domain) (73), HIV (CD4 and co-receptor binding domains) (74), and influenza [extracellular domain of matrix protein 2 (M2e)] (75).

In this investigation, we are not analyzing vaccinated individuals that have a strong response to the correct epitopes. We are studying instead human VL cured and DTH^+^ subjects, trying to detect to what portion of the NH36 molecule the acquired resistance and immunity of these patients is directed. These individuals, although representing the Th1 pole of the immune response to VL, and being expected to have the strongest naturally acquired immune resistance to VL (41–43), display milder immune responses, in *in vitro* assays, if compared to vaccinated subjects. Therefore, the search for immunogenicity is more difficult, and small signals of increase of the immune reactivity should be considered in order to map the best immunogenic domain.

It is worth to note that the predicted epitopes are different in mice and in human histocompatibility complexes. For humans the *in silico* programs disclosed two epitopes for CD3^+^CD4^+^ T cells in F2 and one in F1, and further three epitopes for CD3^+^CD8^+^ T cells in F1 and additional three in F3. In agreement to that, we were able to show that the most pronounced CD4-Th1 response was directed against F2 and F1 domains, and the strongest CD8^+^ response target the F1 and F3 proteins.

In fact, the F2 induced 76% higher frequencies of CD3^+^CD4^+^IL-2^+^TNF-α^+^IFN-γ^−^ than F1, in DTH^+^ subjects, and 100% higher proportions of multifunctional IL-2^+^TNF-α^+^IFN-γ^+^ T cells than F1 and F3, respectively, in cured patients. Additionally, in DTH^+^ individuals, F2 increased the CD3^+^CD4^+^IL-2^+^TNF-α^−^IFN-γ^−^ T cell frequencies 67% more than F1 and 69% more than F3. F2 also enhanced the CD3^+^CD4^+^IL-2^+^ T cell proportions 72% more than F1 and 70% more than F3. Furthermore, F2 was 59 and 87% stronger than F1 and F3 domains, respectively, but most important, it was 96% more potent than NH36, in the enhancement of the frequencies of CD3^+^CD4^+^IL-2^+^TNF-α^−^IFN-γ^+^ T cells of the DTH^+^ subjects.

Accordingly, F2 was the only domain that retained the capabilities of NH36 to enhance the IFN-γ secretion. Additionally, F2 increased the TNF-α secretion 10% more than NH36, and 28% more than the very potent SLA, in cured subjects.

Similar to our results of multiparameter analysis, the increased IFN-γ secretion by PBMC of DTH^+^ subjects, in response to the synthetic epitopes predicted for CD4^+^ T cells disclosed, the F2 (102–121) epitope which promoted a 86 and 60% higher secretion of IFN-γ than the sequences F2 (144–163) and F1 (64–94), respectively.

Regarding the induction of the CD8^+^ T cell response, the reactivity was higher in untreated patients. F1 and F3 increased the frequencies of CD3^+^CD8^+^IL-2^+^TNF-α^−^IFN-γ^−^ T cells above the levels of NH36, by 90 and 89%, respectively. Also, F1 was 59% and F3, 78% more potent than NH36 in the enhancement of the frequencies of CD3^+^CD8^+^IL-2^+^ T cells. F3 was additionally 77% stronger than NH36, in the increase of the CD3^+^CD8^+^IL-2^+^TNF-α^−^IFN-γ^+^ T cell frequencies. Finally, F1 was 99% and F3, 100% stronger than NH36, in the enhancement of the multifunctional CD3^+^CD8^+^IL-2^+^TNF-α^+^IFN-γ^+^ T cell proportions.

Accordingly, the two synthetic predicted epitopes for CD8 T cells of the F1 domain, F1 (20–28) and F1 (92–100), promoted the secretion of IFN-γ of PBMC of DTH^+^ subjects. The F3 epitopes however did not. This is possible due to the fact that the PBMC incubated with the synthetic epitopes belonged to DTH^+^ subjects and not to untreated patients. The analysis should be extended to untreated patients in order to give more information.

We conclude that, in this investigation, the information disclosed by the immunological assays with the domains was mostly supported by the *in silico* predictions. The use of the single immunogenic domains also induced protective effect increments above those promoted by NH36 and/or the other domains. These results will allow us to guide the initiation of the development of a synthetic vaccine against leishmaniasis.

The importance of our findings is enhanced considering three rare achievements (76): (1) we found CD4 Th1-cell epitopes of a CD4 T cell mediated immunosupressive disease; (2) they are highly promiscuous and bind to many HL-DR allotypes; and (3) they are extremely conserved and present in *Leishmania* of two subgenera that cause visceral, cutaneous, diffuse, and mucocutanoeus leishmaniasis.

Previously, all of the vaccine approaches against leishmaniasis have focused on the stimulation of the CD4 T cell response and have neglected the important contribution of CD8 T cells (14). However defined epitopes for both CD4 (LACK) (77) and CD8 (KMP11; GP63, CPB, CPA, Lpg2, histone 3 variant, histone H4)-mediated protective responses against *Leishmania* (78, 79) were described. Recently, it has been proposed that vaccine design will improve through the search for potential candidates that share both CD4- and CD8 T-cell-stimulating capabilities (NH36, A2, P4) (8, 12–14, 26, 80) and through their expression as polytope or poly-epitope vaccines (14, 81). Recently published data indicate that CD8 T cells are very important in protection against *L. major* infection induced by a polytope DNA construct expressing individual MHC-I-restricted peptides in BALB/c mice (82). In that study, the vaccine stimulation of CD8 T-cells resulted in partial protection which was abolished by CD8 T-cell depletion resulting in a predominant Th2 response. This directly confirmed the role of CD8 T-cells in early-stage Th1 response polarization (82). We also previously described that the protection of mice against *L. amazonensis* infection induced by F1 was mediated by CD8 T cells and was abolished by CD8 T cell depletion (12). The present investigation represents a step forward in the definition of the epitopes for CD8 T cells involved in IFN-γ secretion by human PBMC.

Our results confirm the relevance of NH36 in immune regulation in human VL and identified its immunodominant domains and epitopes that could guide the design of a rational and cross-protective T-cell vaccine against human leishmaniasis.

## Author Contributions

MS, FO, DN, and AB conducted the experiments; MS, DN, CB-C, and IP-d-S acquired data; MS, CB-C, MP, CP-d-S, PL, and DR analyzed data; CP-d-S, RA, and PL designed research studies; EC, JM, and AM provided reagents; CP-d-S wrote the manuscript. All the authors have read and approved the final manuscript.

## Conflict of Interest Statement

DN, MP, and CP-d-S are inventors of the patent file PI1015788-3 (INPI, Brazil). MS, IP-d-S, EC, JM, PL, CB-C, AM, DR, and RA declare no conflict of interest.

## Supplementary Material

Figure S1 **Schematic of NH36 and domains fermentation and purification process**. Expression and purification protocol **(A)**. SDS-PAGE (15 %) with Coomassie Blue staining. The slot of the gel showing the run of the NH36 protein is a part of the same gel that has been lined up to be next to the F3 protein. The original gel contained two other proteins between the F3 and the NH36 antigen that were irrelevant to this investigation **(B)**.

Figure S2 **NH36 is a component of SLA**. Serum antibodies of mice vaccinated with NH36, F1, F2, or F3 domains and saponin recognize the SLA antigen of *L*. *infantum* chagasi in an ELISA assay.

Figure S3 **Correlation between the secretion of IFN-**γ** and IL-17 secretion in response to F1 and F2 and the increases in spleen and liver sizes**. The levels of IFN-γ (pg/ml) in response to F1 **(A,B)** and of IL-17 (pg/ml) in response to F1 **(C,D)** and F2 **(E,F)** were assessed in the supernatants of PBMC of patients before treatment (*n* = 4), cured (*n* = 7), and DTH^+^ subjects (*n* = 9) by the Multiplex^®^ MAP-Luminex assay and were correlated to their increases in spleen and liver sizes. Correlation was calculated using the Spearman two-tailed correlation test. *R* and *p* values are summarized in Table 2. Diagonal lines represent linear regression.

Figure S4 **Correlation between cytokines secretion in culture supernatants and clinical and hematological variables**. The levels of IFN-γ and IL-17 (pg/ml) in response to F1 **(A–C)** and F2 **(D,E)** were assessed in the supernatants of PBMC of patients before treatment (*n* = 4), cured (*n* = 7), and DTH^+^ subjects (*n* = 9) by the Multiplex^®^ MAP-Luminex assay were correlated to the hematological parameters. Additionally, we show the correlation between the increases in spleen and liver increased sizes of 41 untreated VL patients **(F)**. Correlation was calculated using the Spearman two-tailed correlation test. *R* and *p* values are summarized in Table 2. Diagonal lines represent linear regression.

Figure S5 **Strategy for the analysis of multifunctional T cell response using a seven-color flow cytometry panel to simultaneously analyze multiple cytokines at the single-cell level in PBMC cultures**. After single cells selection (FSC-A × FSC-H), lymphocytes were selected according to a FSC-A versus SSC-A dot plot, followed by CD3^+^ gating. Afterwards, CD3^+^CD4^+^ and CD3^+^CD8^+^ lymphocytes were evaluated inside the CD3^+^ gate. CD3^+^CD4^+^ and CD3^+^CD8^+^ T-cell phenotypes were plotted against each cytokine individually: tumor necrosis factor-α (TNF-α), interleukin (IL)-2, and interferon-γ (IFN-γ). Boolean gating was performed to generate the frequencies of the possible seven combinations of cytokine producing CD4^+^ and CD8^+^ cells using FlowJo software.
