# Supplementary figures and images for: Leishmania donovani Nucleoside Hydrolase (NH36) Domains Induce T-Cell Cytokine Responses in Human Visceral Leishmaniasis
Source: Front Immunol. 2017 Mar 7;8:227. doi: 10.3389/fimmu.2017.00227 (PMC5338038; doi:10.3389/fimmu.2017.00227)

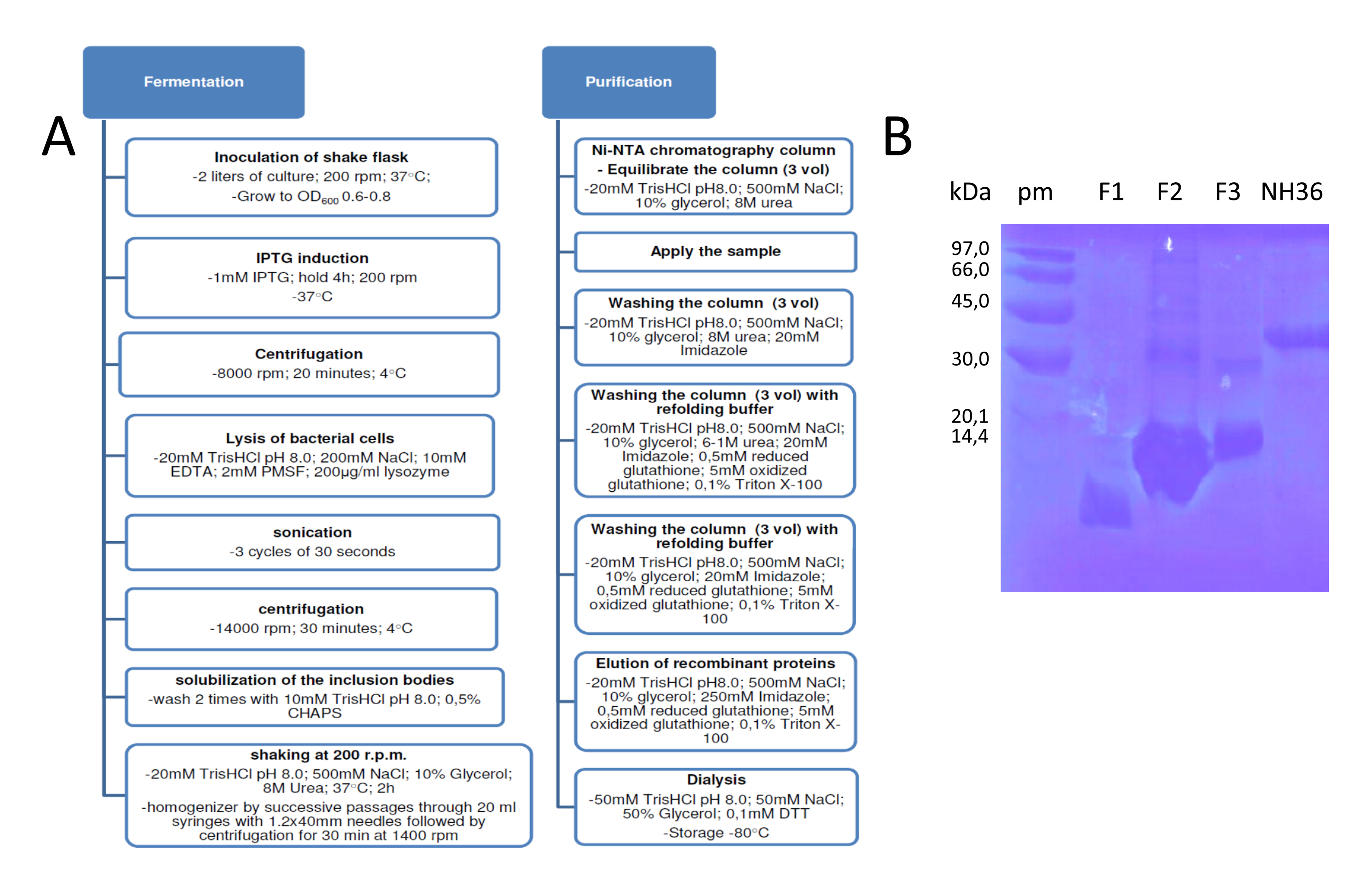

Supplement: Figure S1 — Schematic of NH36 and domains fermentation and purification process. Expression and purification protocol (A). SDS-PAGE (15 %) with Coomassie Blue staining. The slot of the gel showing the run of the NH36 protein is a part of the same gel that has been lined up to be next to the F3 protein. The original gel contained two other proteins between the F3 and the NH36 antigen that were irrelevant to this investigation (B). [file image_1.tif]

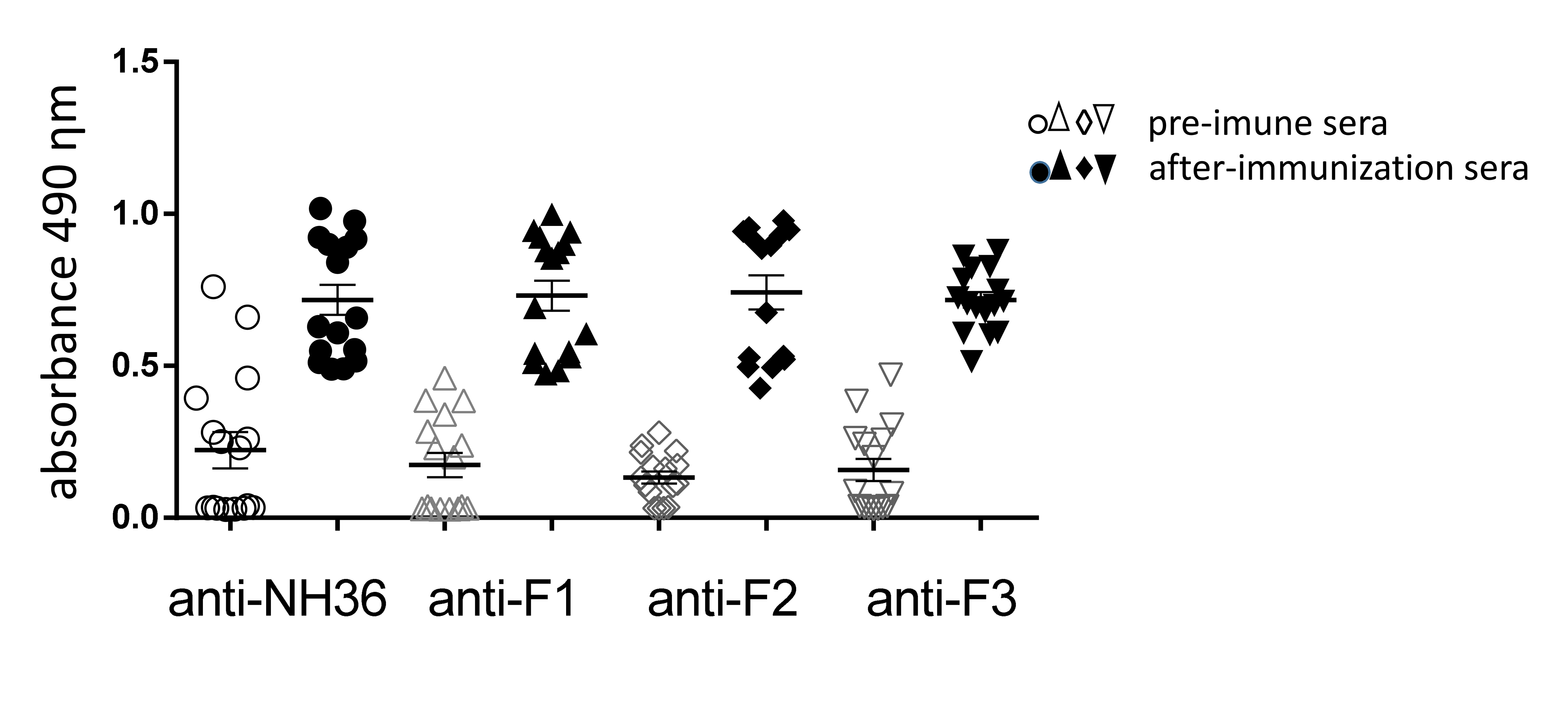

Supplement: Figure S2 — NH36 is a component of SLA. Serum antibodies of mice vaccinated with NH36, F1, F2, or F3 domains and saponin recognize the SLA antigen of L. infantum chagasi in an ELISA assay. [file image_2.tif]

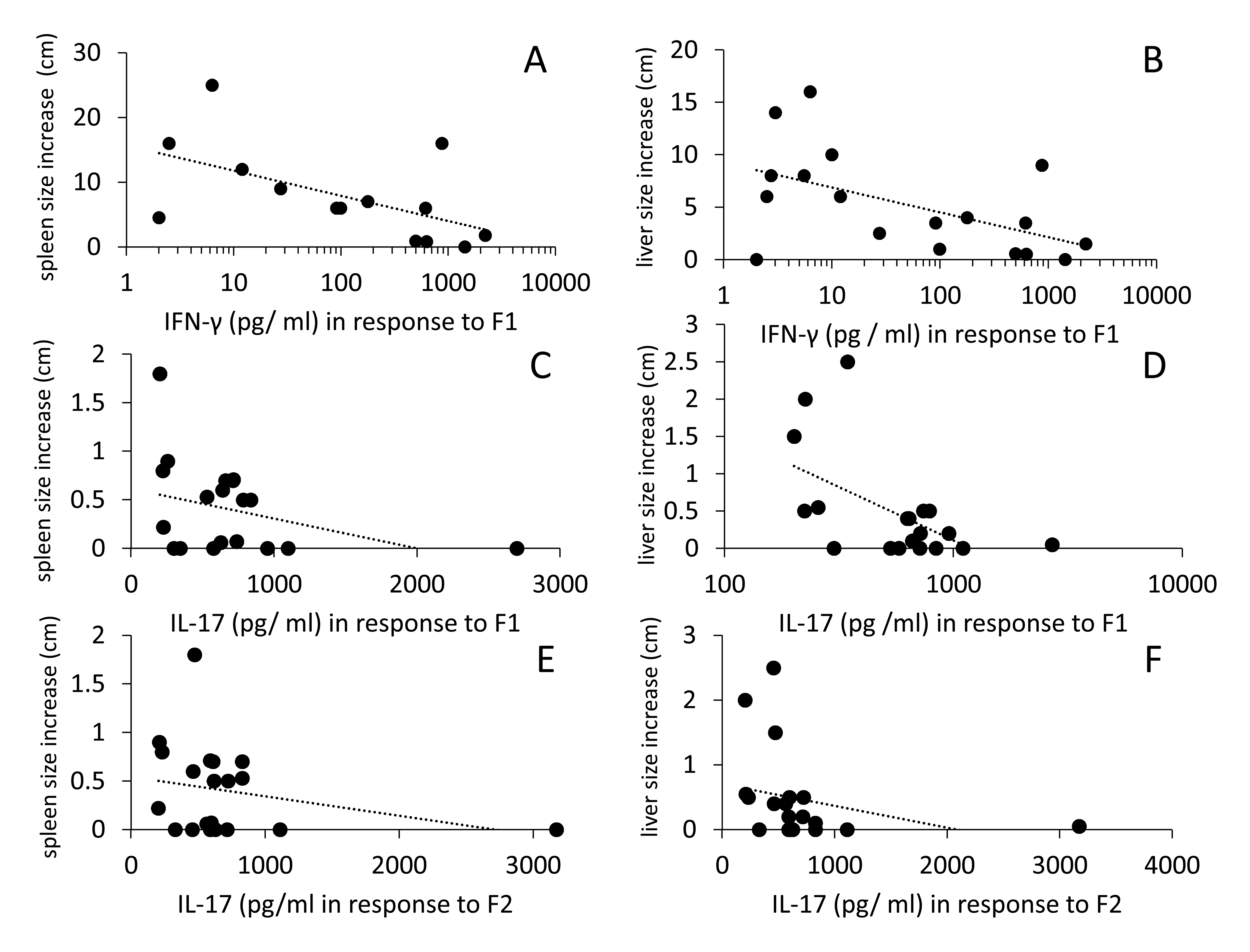

Supplement: Figure S3 — Correlation between the secretion of IFN-γ and IL-17 secretion in response to F1 and F2 and the increases in spleen and liver sizes. The levels of IFN-γ (pg/ml) in response to F1 (A,B) and of IL-17 (pg/ml) in response to F1 (C,D) and F2 (E,F) were assessed in the supernatants of PBMC of patients before treatment (n = 4), cured (n = 7), and DTH+ subjects (n = 9) by the Multiplex® MAP-Luminex assay and were correlated to their increases in spleen and liver sizes. Correlation was calculated using the Spearman two-tailed correlation test. R and p values are summarized in Table 2. Diagonal lines represent linear regression. [file image_3.tif]

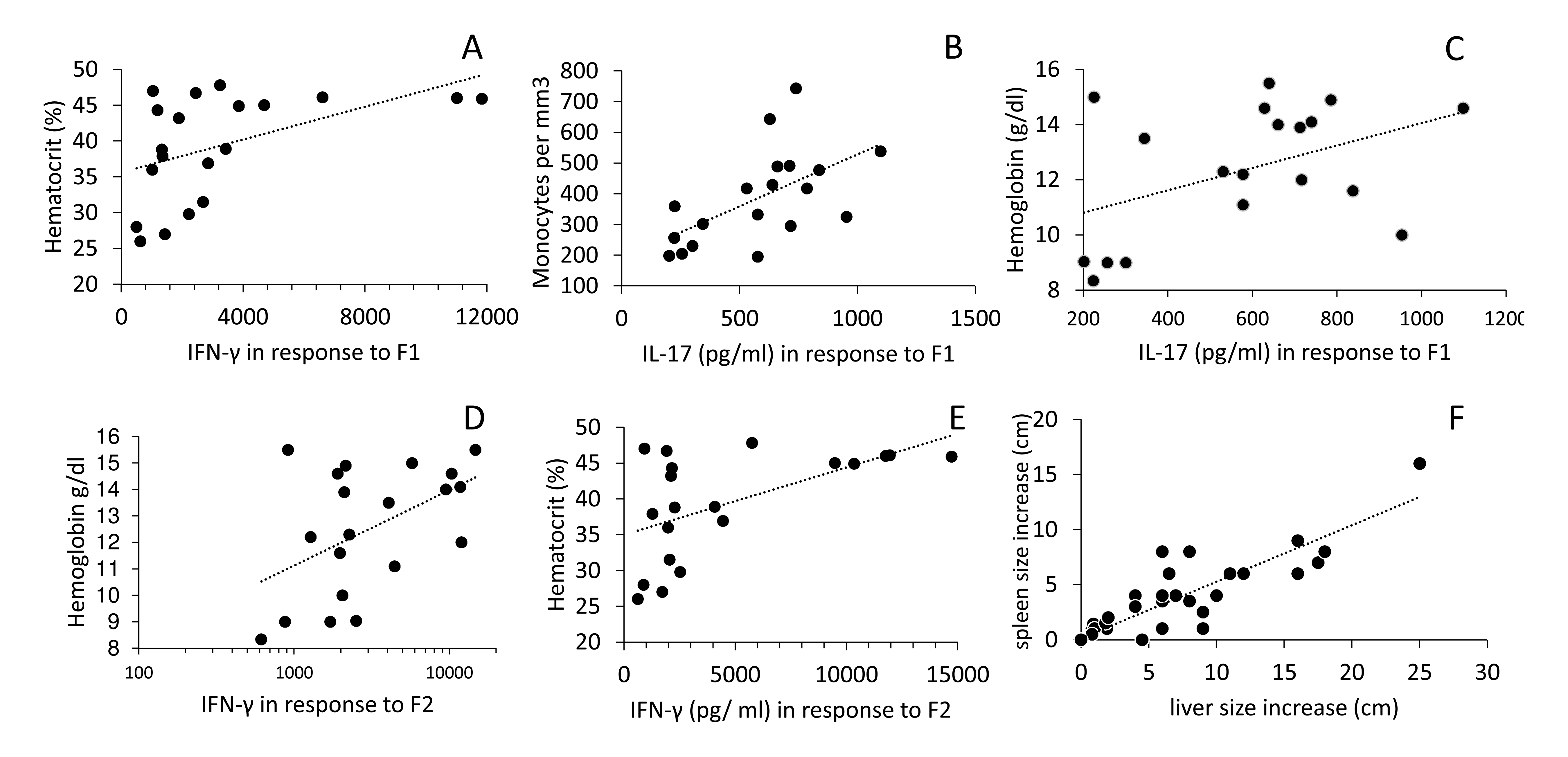

Supplement: Figure S4 — Correlation between cytokines secretion in culture supernatants and clinical and hematological variables. The levels of IFN-γ and IL-17 (pg/ml) in response to F1 (A–C) and F2 (D,E) were assessed in the supernatants of PBMC of patients before treatment (n = 4), cured (n = 7), and DTH+ subjects (n = 9) by the Multiplex® MAP-Luminex assay were correlated to the hematological parameters. Additionally, we show the correlation between the increases in spleen and liver increased sizes of 41 untreated VL patients (F). Correlation was calculated using the Spearman two-tailed correlation test. R and p values are summarized in Table 2. Diagonal lines represent linear regression. [file image_4.tif]

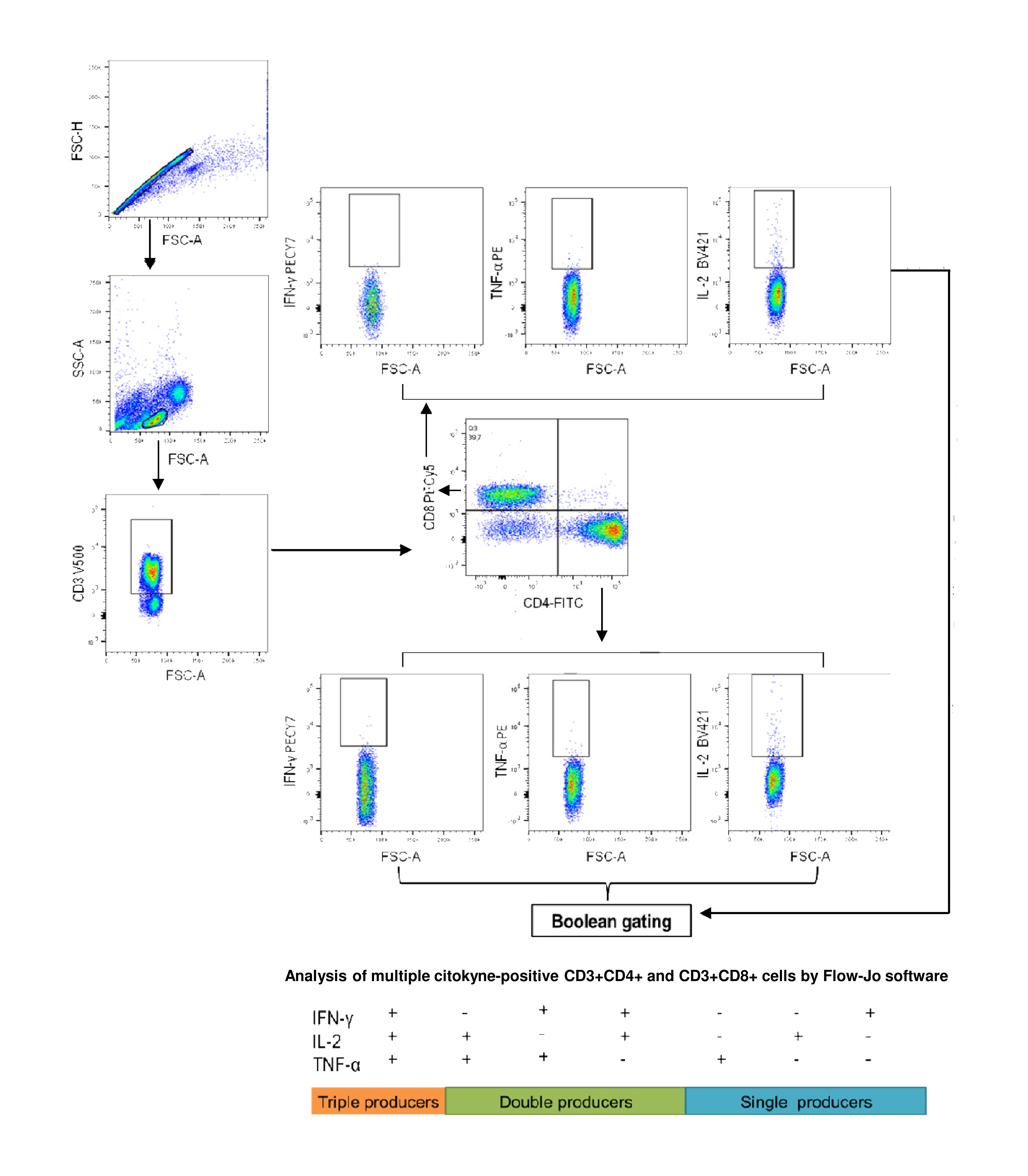

Supplement: Figure S5 — Strategy for the analysis of multifunctional T cell response using a seven-color flow cytometry panel to simultaneously analyze multiple cytokines at the single-cell level in PBMC cultures. After single cells selection (FSC-A × FSC-H), lymphocytes were selected according to a FSC-A versus SSC-A dot plot, followed by CD3+ gating. Afterwards, CD3+CD4+ and CD3+CD8+ lymphocytes were evaluated inside the CD3+ gate. CD3+CD4+ and CD3+CD8+ T-cell phenotypes were plotted against each cytokine individually: tumor necrosis factor-α (TNF-α), interleukin (IL)-2, and interferon-γ (IFN-γ). Boolean gating was performed to generate the frequencies of the possible seven combinations of cytokine producing CD4+ and CD8+ cells using FlowJo software. [file image_5.tif]
